# DyeDactic workflow to predict halochromism of biosynthetic colourants

**DOI:** 10.1038/s42004-025-01881-9

**Published:** 2026-01-10

**Authors:** Dmitry S. Karlov, Rodolfo Marques, Richard J. Wheatley, Jonathan D. Hirst

**Affiliations:** 1 School of Chemistry, University of Nottingham, Nottingham, UK; 2 Colorifix Ltd, Norwich, UK

**Keywords:** Cheminformatics, Computational chemistry, Natural products, Bioanalytical chemistry

## Abstract

Textile dyeing using microorganisms is a step towards sustainable manufacturing. Computational design offers the prospect of new biosynthetic colourants with better dyeing performance, greater photostability, reduced toxicity, and desired colour. We present a workflow (DyeDactic) to predict halochromism, i.e. colour at different pH values. We filter compound libraries using a graph neural network model to estimate the relevant electronic transition energies of potential colourants. The absorption spectra in the visible region and the colours of the resultant molecules are calculated using time-dependent density functional theory. The populations of protonated and deprotonated species are estimated at different pH values. A weighted sum of their computed absorption spectra gives the predicted colour. The DyeDactic workflow is applied to four natural colourants: emodin, quinalizarin, biliverdin, and orcein, followed by experimental validation. As an illustration we also investigated the molecular mechanism of a red to blue colour change when microbial culture containing polyketide bikaverin is autoclaved. The workflow represents a useful tool to guide chemoenzymatic modifications to achieve industrial applicability.

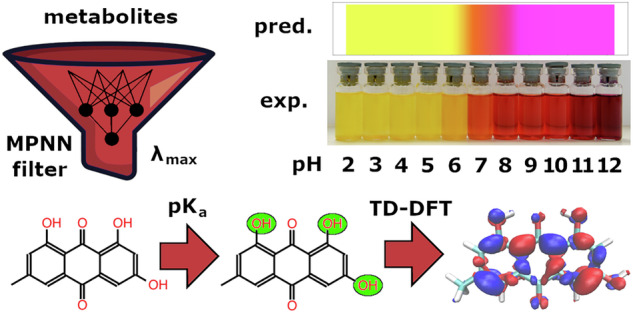

## Abstract (web summary)

Biosynthetic colourants are sustainable alternatives to replace artificial dyes, but prediction of their optical properties remains challenging. Here, the authors develop a workflow combining message passing neural networks and excited state calculations to predict colour changes at different pH levels, offering a promising tool for designing eco-friendly dyes.

## Introduction

Coloured substances have made a huge impact on the development of humanity ranging from the colouration of textiles and food in the early days of civilisation^1^ to recent advances in the design of dye-sensitised solar cells^2^. Photogenerated excited states of dye molecules can facilitate the transfer of their weakly localised electrons to an electrical circuit, a process that results in the production of usable electrical energy. Medical treatment is another non-obvious application of colourants^3^. If a colourant is delivered in a targeted fashion to malignant cells and stimulated by red and near infra-red light, it will generate reactive oxygen species, killing the surrounding cells. This approach is called photodynamic therapy and natural colourants can be used^4^. The traditional dyeing process requires large amounts of water, salts for dyeing of cellulose fibres and polymers for polyester dyeing, making wastewater treatment difficult and contributing to environmental pollution^5^. A more sustainable approach is to use microbially produced dyes^6^. Pigments and microbially produced dyes are subtly different from natural dyes: certain natural colourants can be produced in microorganisms like *E. coli*, but additional enzymatic modifications in a controlled fermentational environment can lead to molecules which have not been isolated as natural products. Applying microbially produced dyes directly in a cultural liquid as a dyeing fluid consumes less water and does not employ petrochemical derivatives, mitigating environmental hazards. However the transition to this dyeing process will require highly-qualified staff and capital investment to install fermentation equipment. Challenges include the notorious photoinstability of natural colourants^7^ and a difference in structural and physicochemical properties between synthetic and biosynthetically produced dyes and pigments.

The principles and approaches of synthetic biology^8^ which utilises computational modelling, can be applied to overcome these challenges. In the pharmaceutical industry^9^, in silico approaches have made design processes faster and have reduced costs. Enzymatic modifications started to appear in, for example, the food industry producing flavour enhancers^10^ or sweeteners^11^ using glycosylation, methylation, or prenylation tailoring steps to eliminate undesirable properties like bitter taste. In recent years many biosynthetic pathways of natural colourants were cloned in heterologous hosts, reaching high concentrations of colouring matter in cellular culture (blue pigment indigoidine^12^, green pigment phycocyanobilin^13^, etc.). To be accepted in industry the modelling of the optical properties of colourants must be fast and reliable and be used in conjunction with an efficient tool to explore the available chemical space. Machine learning (ML) approaches are used in many industries to make decisions quickly based on the available data^14^, including self-driving cars and equipment, financial forecasting and analysis of medical data. Nevertheless ML predictions are usually less accurate on unseen data. So uncertainty estimation is crucial for decision-making based on ML predictions.

In this work we collated a representative database of natural colourants. We identified structural features of natural colourants and their similarity to different classes of artificial dyes. The corresponding optical properties of the natural colourants were used to fine-tune an ML model and to choose an appropriate approach (TD-DFT^15^) for quantum chemical calculations, balancing computational cost and accuracy. In contrast to artificial colourants, which typically do not exhibit halochromicity close to neutral pH, the colour of natural and biosynthetically accessible colourants can depend on pH. Thus, an important final step of the workflow estimates the pH-dependence of colour, by considering populations of protonated and deprotonated molecular species and their predicted absorption spectra. The predicted colour vs. pH plots were validated against experimental data produced for a set of four natural colourants spanning different classes of organic compounds. The four halochromes are readily (and cheaply) available from many vendors. They include both rigid and flexible molecules, absorb light in different regions of the visible spectrum and they have different functional groups which can be protonated and deprotonated at different pH values.

We also illustrate the applicability of the workflow by considering polyketide bikaverin^16^, which exhibits an unexpected colour change, from red to blue, when the culture is autoclaved. We explored the chemical space around the parent molecule, predicted the colour of derivatives and showed that amination, which can easily happen to naphthoquinones^17^ upon heating, will cause a blue colour even at the mildly acidic pH used for bikaverin production.

We have developed a multilevel workflow combining ML for the rapid screening of potential colourants and more robust, but computationally demanding, quantum chemical calculations for the identification and subsequent refinement of the predicted colour. The computational platform is flexible and readily extensible to the study of optical properties, including fluorescence, photoreactivity, catalysis and electroluminescence.

## Results and discussion

### Exploratory analysis of natural colourants

**Fig. 1:**
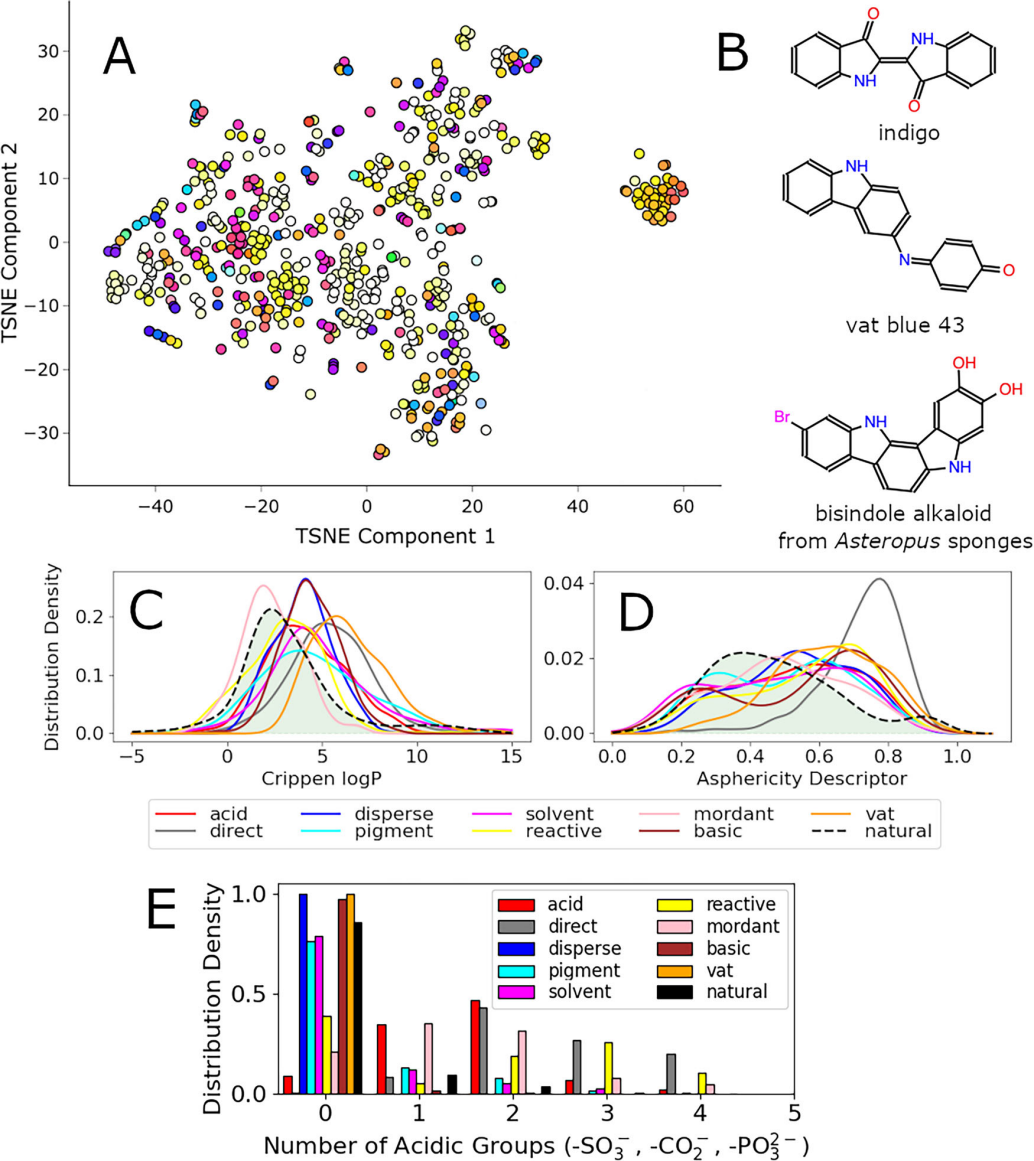
Characteristics of the database. **A** T-SNE visualisation of the collected natural colourants database with each point representing a colourant. The colour of each point was calculated based on the experimental information about the wavelengths of absorption maxima. **B** Examples of vat dyes and a related natural product. Visualisation of dyeing-related molecular properties distributions: lipophilicity (**C**) and asphericity descriptor (**D**) and number of acidic groups (**E**), respectively. The comparison is made between industrial dyes from the Colour Index (solid lines) and the collected natural product database (black dashed line, area under the distribution shown in green). Source data underlying the graphs and charts presented in all the figures are provided in the Supplementary Data.

**Fig. 2:**
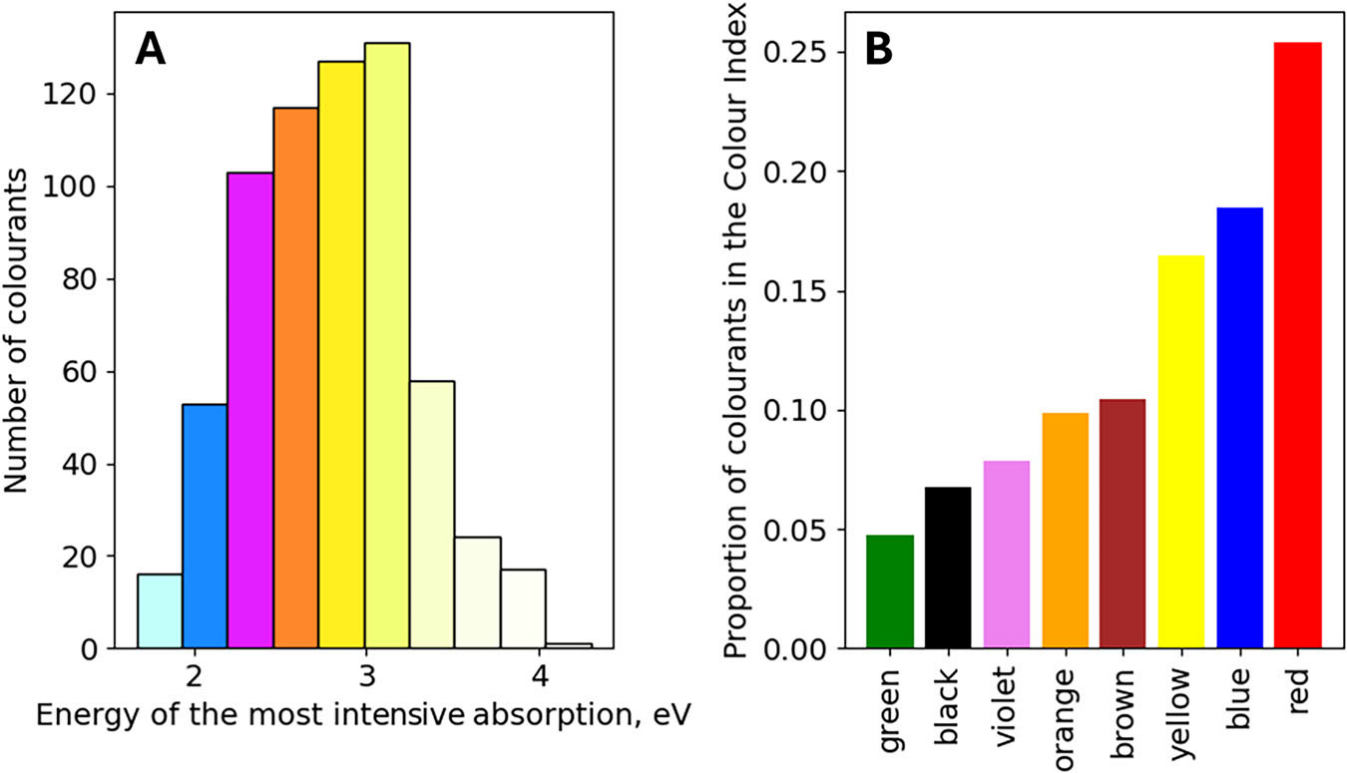
Distributions of electronic transition energies and colours. **A** The distribution of energies of the most intense light absorption in the natural colourant database. Bar colours are derived in the following way: energy values from the centre of each bar are taken for further processing; a Gaussian with *σ* = 0.2 eV is centred at this point on the energy scale and the obtained approximation of the spectrum is converted to RGB as described in the ‘Methods’ section. **B** Colour information taken from colourant names from the Colour Index (colour index names follow a pattern: ‘class colour number’, for example, vat blue 1 states for indigo).

Our assembled database of natural colourants with their corresponding spectroscopic properties consists of 647 compounds. These compounds belong to 39 classes (Fig. S1), based on their chemical nature (anthraquinones, flavonoids) or their biosynthetic precursors. A t-SNE^18^ visualisation of the chemical space is shown in Fig. 1A and contains multiple clusters of related compounds; the cluster on the right of the plot is composed of carotenoids. The colour of each point is derived from experimental spectroscopic properties of the compound and can vary strongly with respect to minor structural changes. Similar compounds like indigoids demonstrate a drastic colour change from yellow to blue and green. The populations of green, blue and purple substances, which absorb longer wavelengths, are lower compared to the other coloured substances and this observation is confirmed by the absorption energy histogram (Fig. 2A). The scarcity of blue metabolites^19^ is a hurdle for the delivery of stable natural colourants for industrial application. Conversely, blue synthetic dyes and pigments from the Colour Index (a database of synthetic dyes used for comparison) are the second most frequent colour after red (Fig. 2B). Thus the structural fine-tuning of natural colourants is possible and can deliver desired colour changes.

Although natural colourants can boast a good absorption coverage of the visible spectrum, a bright colour is not enough for textile dyeing. To investigate which features are important a comparison of structural and physicochemical properties was performed between our collected natural colourant data set and different classes of dyes and pigments from the colour index. The distribution of molecular size of natural colourants has a bimodal character with the main maximum located near 21 atoms and a second smaller peak near 42 atoms (Fig. S2A). The first peak reflects the average size of coloured molecules; it is shifted to lower values of molecular weight than peaks from artificial colourant classes. The second peak is related mainly to dimeric and oligomeric pigments which are formed from two pigment building blocks during the biosynthetic process. Lipophilicity is a principal property for dyeing of hydrophobic materials (polyester and acetate fabrics). Natural colourants are more hydrophilic compared to most of the dye classes (Fig. 1B) and the distribution is similar to mordant dyes, which stick to some fabric types because of metal-assisted coordination interactions. Taken together with the low number of easily ionisable groups (Fig. 1D) this leads to the observation that the hydrophobicity distribution of natural colourants is shifted due to polar non-ionisable groups like hydroxyls or carbonyls. To make a natural colourant more suitable for dyeing of hydrophobic materials in fine dispersion and to improve lipophilicity, modifications like the addition of a hydrophobic tail can be considered. A distinct feature of industrial disperse dyes is a larger fraction of rotatable bonds compared to other colourants (Fig. S2C).

Two classes of artificial colourants have the greatest difference from natural colourants in terms of structural properties: direct and vat dyes. Direct dyes are large molecules with extended shape, which is confirmed by the shift of their asphericity ^20^ distribution towards one (Fig. 1C). Their affinity to cotton is explained by hydrophobic interactions with amorphous regions of the cellulose fibre^21^. Dimerisation is a way for biosynthetic colourants to reach the dyeing capabilities of this class of compounds. Vat dyes are large polycyclics, usually composed of six to ten cycles (Fig. S2B), with a low fraction of rotatable bonds (Fig. S2C). Conversely, the reduced form is soluble in the dyebath and gradual oxidation by oxygen deposition of dye aggregates on fibres. Indigo is among a few natural colourants used in a vat process^22^ long before synthetic dyes (Fig. 1E). However it is not the only option: for example, the bisindole alkaloid isolated from sea sponges^23^ is similar to vat blue 43 dye from the Colour Index (Fig. 1E). This bisindole is also blue probably due to the presence of oxidation products, making it an interesting starting point for development of nature-derived vat dyes. The likelihood of a colourant belonging to a particular dyeing class is estimated based on a set of simple descriptors mentioned above and is rapidly calculated as a part of the colour prediction workflow. Based on this analysis we conclude that natural colourants from the collected database are not particularly similar to any of the artificial dyeing classes and additional biosynthetic modifications should be carried out to develop colourants with dyeing properties suitable for industrial applications.

Light absorption at a longer wavelength which causes a substance to be blue, requires a more extended conjugated *π*-system with bound electron-donating heteroatoms. Statistical analysis shows nitrogen-containing compounds from the data set tend to have significantly lower absorption energies by 0.13 eV on average compared to colourants not containing nitrogen (Mann–Whitney ^24^ two-sided test, *P* = 0.0031). The absorption energy has also a weak, yet significant, inverse correlation with the size of the *π*-system calculated as a longest path composed of carbon and nitrogen atoms in an *sp*^2^-hybridisation state (Pearson *r* = −0.20, *P* = 2.2 × 10^−7^). These two observations explain the strong structural influence of small perturbations in colourant structure and the accuracy of colour prediction by quantum chemical calculations, which model molecules holistically.

### Performance of transition energy prediction techniques

**Fig. 3:**
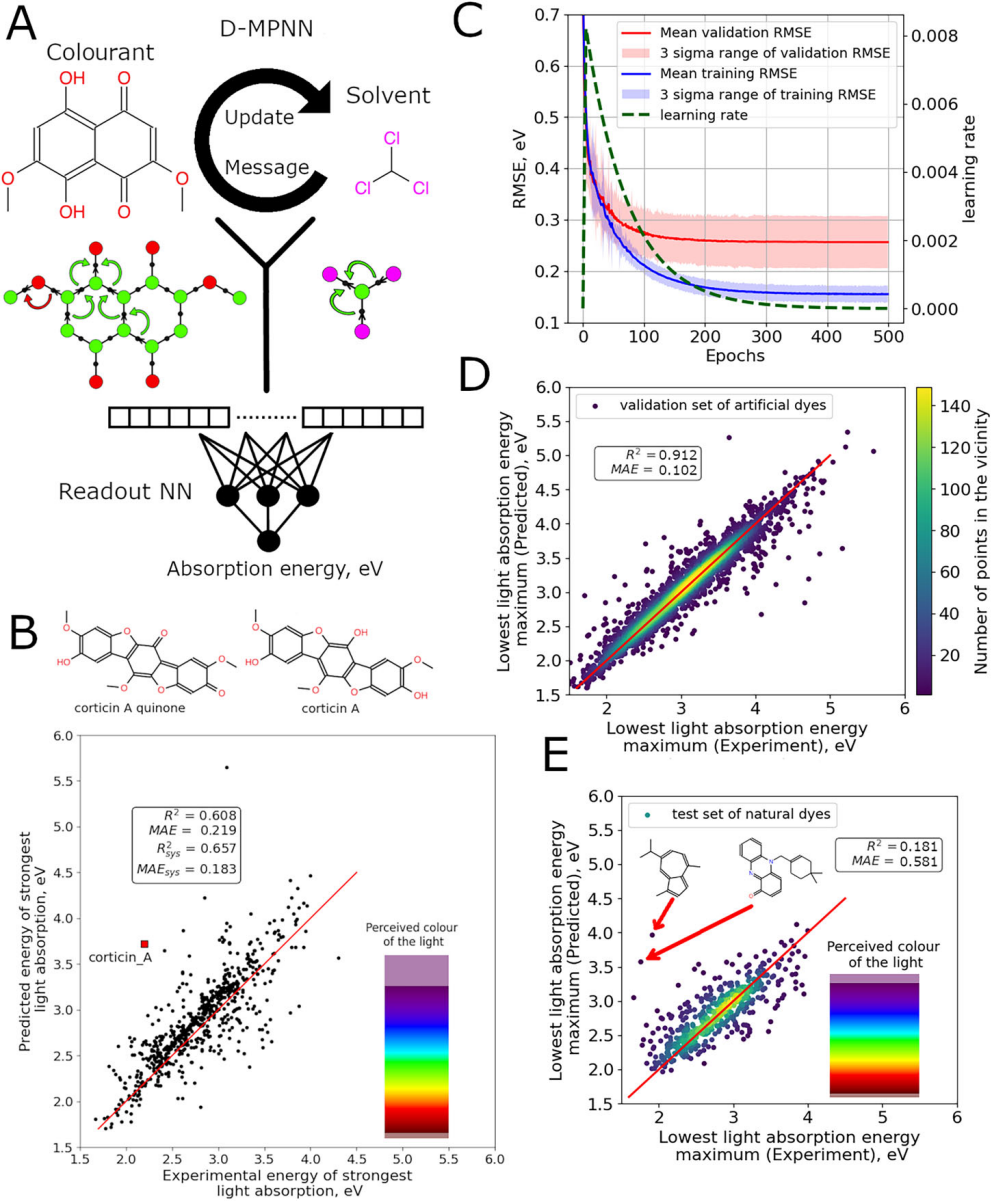
Machine learning approach and performance. **A** MPNN approach to predict λ_max_ for colourants taking into account solvent and colourant structure. **B** A parity plot between calculated lowest vertical transition energies for the natural product data set. Systematic correction is not applied and the red line is the bisector illustrating perfect correlation. ‘Perceived colour of light’ palette provides a correspondence between the light of the given energy and its colour. **C** Neural network loss changes during training. Training and test set losses are shown in blue and red, respectively. Green dashed line marks learning rate changes over training (axis on the right belongs to it). Training and validation error uncertainties were estimated based on five-fold cross-validation (transparent blue and red). **D** Prediction results for the validation set composed of artificial dyes (yellow to purple; yellow marks the highest density for points) and **E** prediction results for the natural colourants test set, the adopted colour scheme is the same as in (**D**). The colour bar on the right shows the perceived colour of light with energy on the y-axis. Structures of the two outliers are shown.

Based on the exploratory analysis, we consider two ways to develop generalised approaches to predict light absorption energy and consequently colour: ML methods like MPNN^25,26^, which support information flow across the molecular graph (Fig. 3A) or simpler approaches together with descriptors derived from quantum chemical calculations (e.g. HOMO-LUMO gap). The MPNN approach considers structures of both colourants and solvents which are visualised in a graph format with coloured circular nodes and edges (Fig. 3A). Arrows schematically show information flow during the ‘message’ and ‘update’ phases. The final phase includes mapping of the descriptors into a fixed-length vector and an application of conventional neural networks to predict a property of interest.

A model for maximum absorption wavelength prediction based on the MPNN canvas achieved an impressive performance for artificial dyes^27^, but was much less accurate (MAE > 0.3 eV) for the collected set of natural colourants (Table 1). The accuracy was comparable to the baseline model (MAE = 0.31 eV) using just a single HOMO-LUMO gap descriptor provided by GFN2-xTB calculations^28^ (Table 1). There are two possible reasons for this behaviour: limited coverage of natural colourant classes by the artificial colourant training set used in the original model and the quality of the collected spectroscopic data from the natural colourant data set. To ensure the high quality of the added data, excited state calculations (TD-DFT) were carried out to detect outliers, which can seriously affect the model performance.

**Table 1:** Performance of baseline, D-MPNN, combined ML + QM and TD-DFT approaches to calculate transition energies (in eV)

ID	Method		*R*^2^	MAE	*R*^2^ (sys. corr.)	MAE (sys. cor.)	*R*^2^ (sys. corr.)^c^	MAE (sys. cor.)^c^
1	Baseline HOMO-LUMO gap (GFN2-xTB)		−6.27	1.271	0.277	0.308	–	–
2	Application of a published D-MPNN trained only on artificial colourants		0.181	0.321	0.324	0.267	–	–
6	D-MPNN trained using the collected natural colourants^a^		–	–	–	–	0.581	0.184
3	Linear regression + QM descriptors^b^	⍵B97X-D4	–	–	–	–	0.443	0.191
4		⍵B97X-D4 + CPCM	–	–	–	–	0.441	0.191
5	TD/TDA-DFT calculation	PBE0	0.430	0.278	0.504	0.244	0.650	0.201
6		PBE0 + CPCM	0.450	0.263	0.509	0.247	0.653	0.205
7		⍵B97X-D4	−0.798	0.615	0.619	0.209	0.766	0.166
8		⍵B97X-D4 + CPCM	−0.302	0.514	0.630	0.208	0.776	0.164
9		⍵B97X-D4 + TDA	−1.79	0.779	0.600	0.214	0.754	0.169
10		⍵B97X-D4 + TDA + CPCM	−1.01	0.659	0.623	0.210	0.772	0.164
11		BMK	0.037	0.423	0.569	0.227	0.727	0.181
12		BMK + CPCM	0.290	0.364	0.577	0.229	0.724	0.184
13		CAM-B3LYP	0.247	0.347	0.566	0.226	0.727	0.180
14		CAM-B3LYP + CPCM	0.434	0.299	0.579	0.227	0.728	0.182
15		M06-2X	−0.11	0.46	0.596	0.217	0.757	0.172
16		M06-2X + CPCM	0.204	0.382	0.607	0.217	0.759	0.171
17		B2PLYP	0.461	0.262	0.553	0.222	0.717	0.175
18		B2PLYP + CPCM	0.551	0.220	0.593	0.212	0.757	0.163
19		SCS-PBE-QIDH	0.269	0.346	0.597	0.207	0.761	0.159
20		SCS-PBE-QIDH + CPCM	0.518	0.255	**0.657**^d^	**0.183**	**0.822**	**0.133**
21		SCS-⍵PBEPP86	0.404	0.304	0.603	0.206	0.764	0.158
22		SCS-⍵PBEPP86 + CPCM	0.605	0.220	**0.657**	**0.183**	**0.816**	**0.135**

Determination coefficient (*R*^2^) and mean absolute error (MAE) are used to rank the performance of the approaches before and after the removal of systematic error components. The last two columns on the right are used to compare the ML model trained on the cleaned dataset obtained based on the five-fold cross-validation and TD-DFT calculation results.

^a^Calculated in cross-validation for a cleaned natural colourants dataset containing 595 compounds versus 647 molecules in the original set.

^b^10-fold CV.

^c^Dataset with the outliers removed which was used to train the ML models.

^d^Bold font marks the best performing techniques.

The performance of the explored functionals is shown in Table 1. The error observed for each functional can be split into systematic and random components and the functionals are ranked based on the latter. The Tamm–Dancoff approximation^29^ (TDA) does not affect performance in this case. The application of CPCM^30^ solvation dramatically improves performance on determination coefficients before the systematic error correction and MAEs, but after correction, only double hybrid functionals demonstrate a noticeable improvement. The recently developed approaches SCS-⍵PBEPP86 and SCS-PBE-QIDH^31^ (Table 1) provide the best estimation of transition energy. However, unfavourable scaling limits their applicability to relatively small molecules. The much less demanding ⍵B97X-D4^32^ functional gives the best accuracy among non-double hybrid functionals after the removal of systematic error (Table 1).

The prediction errors are strongly affected by outliers (Fig. 3B). An important aspect of the data set is that a significant number of natural substances were isolated and purified only once and there are no other works confirming the identity or purity of the colourants. For example corticin A^33^ (Fig. 3A) is a blue solid (*E*_exp_ = 2.19 eV) whose colour is explained by the presence of oxidation products. Calculation of a putative corticin A quinone (Fig. 3A) yields several intense transitions with *E*_vert_ = 1.92 eV (after systematic error correction of single-point TD-DFT calculations using the ⍵B97X-D4 functional with the def2-TZVP basis set with the CPCM solvation model and geometry optimised with the PBEh-3c composite approach and the CPCM solvation model), supporting the possibility of a blue colour in quinoid form.

Strong correlation between experimental and calculated transition energies for natural colourants and the relatively low number of outliers (~10% with MAE > 0.5 eV) warrants the use of the collected natural colourant data set to augment the initial training set^34^ used by Greenman et al.^27^ and retrain the model (Fig. 3C–E). This modification improves prediction accuracy (MAE = 0.18 vs 0.32 eV and *R*^2^ = 0.58 vs 0.18 for models out of the box, Table 1). Despite the improvement in the overall performance of the D-MPNN model^26^, several outliers have been detected. This includes azulene derivatives which are not well represented in the training set due to the scarcity of spectral data in the literature. The speed of the prediction process makes it possible to use the model for a fast pre-screening of potential colourants with subsequent accurate TD-DFT calculations.

An explanation for a strong divergence between computed and experimental transition energies in several cases (*E*_calc_ « *E*_exp_) may be the low extinction coefficients of the corresponding absorption bands. Accurate estimation of extinction coefficients (log_10_ε) is a complex task, because of numerous sources of error^35^ and it is especially hard for compounds absorbing visible light^36^. For example, the MAEs of extinction coefficients (0.38) obtained from oscillator strengths using simple linear regression (Table 1) are comparable for predictions using ML on the Deep4Chem data set^34^ (0.37), yet no significant correlation is observed between oscillator strengths and molar extinction coefficients for the whole database of natural compounds (Table 1).

Excited state calculations require more time than those for the electronic ground state and accuracy improves upon the removal of a systematic error using a ML treatment (Table 1). A set of descriptors^37^ (HOMO and LUMO energies, hardness (η), dipole moment, etc.; Table S5) is calculated for the ground state of natural colourants using a long-range corrected hybrid density functional with an intrinsic dispersion correction—⍵B97X-D4, employing a sufficiently large basis set def2-TZVP with solvation effects taken into account by the conductor-like polarised continuum model (CPCM) and linear regression is applied (Tables S6 and S7). With respect to prediction error, this approach does not have an advantage (Table 1 and Figs. S3–S5) over the most reasonable TD-DFT approach (⍵B97X-D4/def2-TZVP (CPCM)//PBEh-3c (CPCM)) and the latter directly provides information on high energy transitions, which improves colour estimation.

### Halochromic properties prediction and experimental validation

**Fig. 4:**
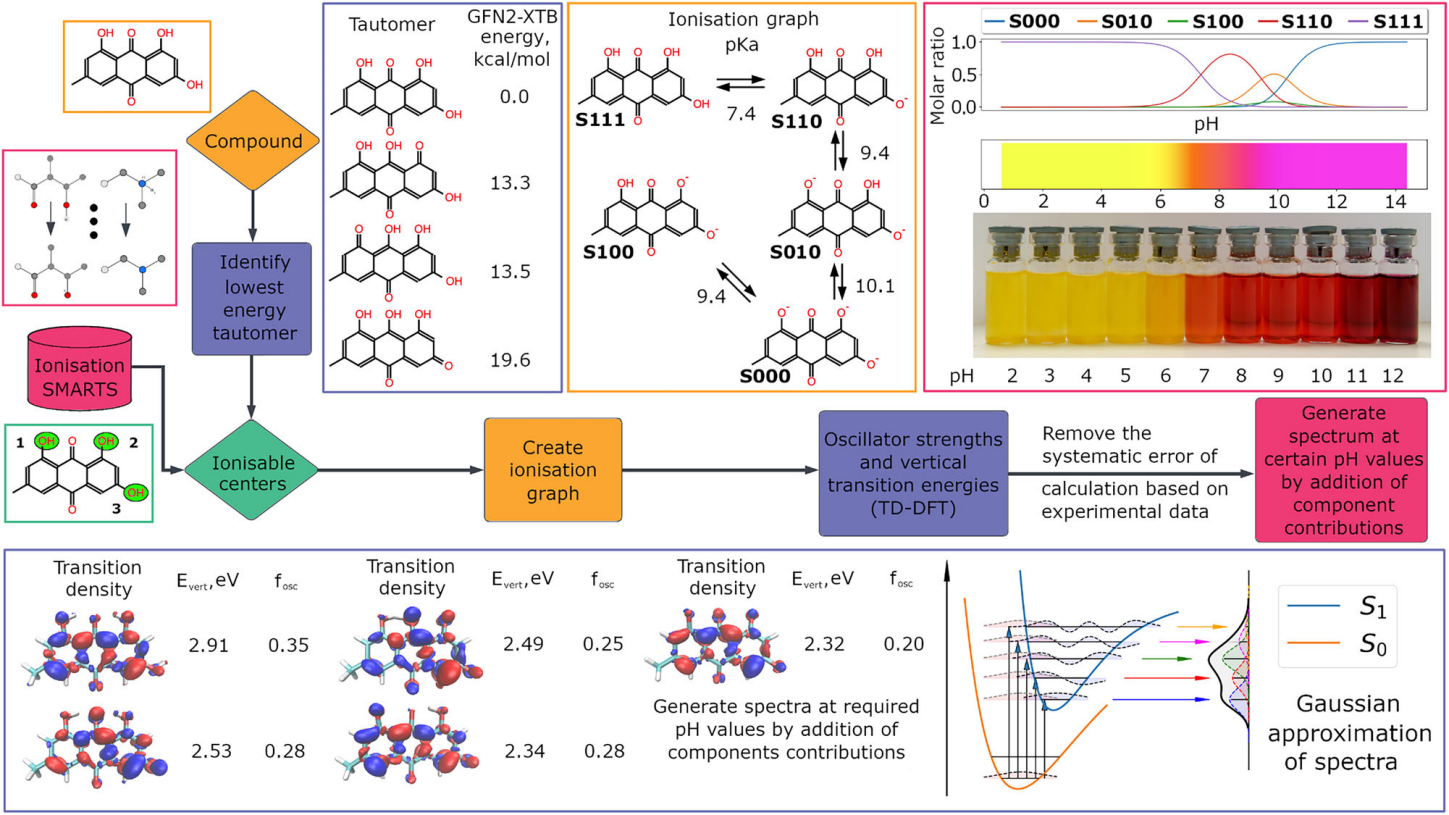
DyeDactic colour vs. pH prediction workflow applied to the natural anthraquinone dye emodin. The first step is identification of ionisable groups followed by prediction of acidity constants by a pKa prediction tool (Chemaxon in this case). Protonated species are identified and their molar ratios vs pH are generated. The ionisation graph does not include species with the largest molar ratio below 0.01. Excited state calculations are done for all ionised species and UV–vis spectra are approximated by a simple Gaussian after the systematic error removal or considering vibronic transitions. Schematic vibronic transitions from the singlet *S*_0_ ground state to the first singlet excited state *S*_1_ are shown as illustration.

Natural colourants usually have different absorption spectra depending on the pH of the media and all compounds having protonatable or deprotonatable groups can undergo a colour change. On the one hand this makes them useful indicators^38^, but on the other hand, the colour of a natural substance can be considered only given the pH of the media. Additionally different textile dyes tend to bind different forms of colourants: neutral (e.g. polyester) or charged (protein fibres), which have a significant impact on the colour of fabric. Taking into account only protonation/deprotonation events, the pK_a_ values were predicted using the ChemAxon pKa Predictor, populations of charged species generated and the corresponding absorption spectra calculated, scaled and added, providing a colour estimation at any given pH. Figure 4 contains an example of the colour prediction workflow for a plant metabolite emodin. pH changes cause reversible deprotonation of chromophore-attached hydroxyl groups, contributing to a bathochromic spectral shift. The strength of the shift increases with the degree of deprotonation (Fig. 4, transition energies for deprotonated emodin species), which is observed in both experiment and calculation. The predicted magenta colour (rather than red) of the fully deprotonated emodin can be observed in less concentrated solutions, although it is hard to work with them because colour fading due to oxidation happens quickly at high pH.

The workflow was applied to three other challenging substances: quinalizarin, orcein and biliverdin, which belong to different classes of natural colourants (Table S8). Quinalizarin has a different distribution of hydroxyl groups compared to emodin causing a dramatic colour change from orange to purple, then to blue at highly basic pH values. This is perfectly captured by the colour prediction approach (Fig. S7). The predicted colours of emodin and quinalizarin (Fig. 4 and Fig. S7) are slightly different from the experimental ones derived at acidic pH (Fig. S9 and Table S9). This effect is due to the scattering of light on pigment particles, which have low solubility in a fully protonated state in a water/methanol environment. However the colour at basic pH is quite close to the experimental observations (Table S9). The third pigment orcein is a mixture of compounds^39^ with unknown composition, which represents a major challenge. The colour prediction for this substance considered only two components for simplicity: α-hydroxyorcein (Fig. S8A) and α-aminoorcein (Fig. S8B). Each component alone does not explain the experimental results, while the colour change of the 1:1 mixture is very similar to the experimentally observed results (Fig. S8C).

Experimental peak shapes for emodin, quinalizarin and orcein demonstrate asymmetry and fine structure of absorption peaks, due to transitions to vibrationally excited levels of the electronic excited state. Modelling was carried out to test if there is any improvement of colour prediction when vibronic transitions are taken into account. In general, there was no advantage for colour prediction (Fig. S10). Application of systematic error correction causes a red shift to the transition energy of the negatively charged species, but when the systematic error correction is absent, the spectra of neutral anthraquinones demonstrate a strong blue shift and the predicted colour is incorrect.

**Fig. 5:**
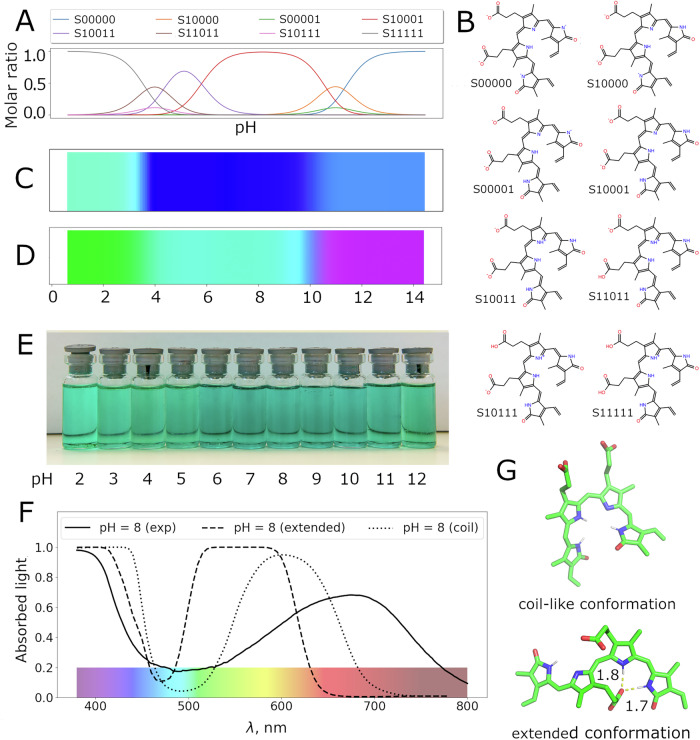
Colour prediction for biliverdin in water and comparison with experimental data. **A**, **B** Populations and structures of biliverdin protonated forms depending on pH. **C** colour prediction of biliverdin in implicit water based on a single optimised conformation. **D** Ensemble-based colour predictions for biliverdin in explicit water with 0.1 M sodium chloride. **E** Experimental biliverdin colour dependence on pH. **F** A comparison of biliverdin spectra: experimental at pH 8 which predominantly contains the S10001 form, ensemble-based colour prediction based on the coil-like conformation of S10001 and its extended conformation stabilised by hydrogen bonds. The amount of absorbed light is given as a proportion and varies between 0 and 1. **G** Representative structures of the extended and coil-like conformations.

The haem degradation product biliverdin represents a different challenge due to its flexibility. Since only one conformation is used by default for colour prediction, the results depend on the choice of conformation. The predicted green colour for the fully protonated form is very similar to what is observed experimentally in an acidic environment (Fig. 5C and Table S9). However the optimised conformations of the partially deprotonated species have higher oscillator strengths for the red light absorption band (Table S8) compared to the blue light band, which causes blue and cyan to be predicted as the colours of the partially deprotonated species; this is different from experimental observations. Their twisted conformations have a lower degree of overlap in the conjugated subsystem causing a hypsochromic spectral shift and carboxylic groups in deprotonated forms of biliverdin tend to maintain hydrogen bonds with pyrrole rings in optimised structures in implicit solvent. If the calculation is done for an ensemble of conformations in explicit water and ions, carboxyl will not form intramolecular hydrogen bonds so readily as they have ionic partners in solvent; this approach may improve the prediction of absorption peak intensity and location.

To test this hypothesis replica exchange molecular dynamics (REMD) simulations with subsequent TD-DFT calculations were carried out. As expected, this improved colour estimation of protonated forms existing at low pH (S11111, S11011, S10111, S10011, S10001), which all are predicted to be a shade of green when an ensemble of conformations was considered (Fig. 5, panel A, B, D). Initial conformation generation for protonated forms of biliverdin led to an extended conformation of the chromophore (Fig. 5G), which in the REMD simulations underwent a rapid transition to a coil-like conformation for chromophores S11111, S11011, S10111 and S10011. This coil-like conformation is observed experimentally^40^ and, due to a higher degree of conjugation, absorbs light with a longer wavelength (Fig. 5F). However initially S10001 did not convert to the stable coil-like structure during the replica exchange simulation and was trapped in the extended conformation, causing a hypsochromic shift of the absorption maxima. To test the hypothesis that the barrier of transition is too high for the tested conditions, the REMD simulation for S10001 form was restarted from the coil-like conformation. In this case the ensemble-based prediction led to the greenish-blue colour observed experimentally. The biliverdin forms with deprotonated amide groups (S10000, S00001, S00000) likely exist at much more basic pH levels, since NMR data^41^ do not demonstrate any conformational change at pH 10.5 and these forms do not influence the colour of biliverdin solution at experimental pH and the predicted spectra should correspond to different shades of green at all pH. This observation emphasises the importance of an accurate estimate of acidity constants.

The DyeDactic workflow focuses on properties related to textile dyeing: colour dependence on pH and structural features contributing to fabric affinity. It can be extended and fine-tuned for identification of biosynthetically accessible compounds exhibiting valuable optical properties like fluorescence. If spin-orbital coupling is estimated between the first excited singlet state and triplet states with lower energy, the workflow can be applied to the discovery of photoreactive compounds with medical application, or to the design of photoredox catalysts. Use of an appropriate method to take into account double excitation effects like CCSD will allow one to fine-tune more sophisticated properties, such as the singlet/triplet energy gap and its sign, which is important for electroluminescence applications.

### Workflow application: bikaverin case

As an example of colour prediction our workflow is applied to a generated set of bikaverin analogues. Bikaverin is a red polyketide pigment with anticancer properties; the colour of the culture turns blue when autoclaved^16^ and the blue colour cannot be extracted with ethylacetate, indicating its potential association with membrane or proteins. There are also non-related polyketides with a blue colour reported (anthracyclinones blue A and B^42^), which results from a non-enzymatic reaction of the anthracyclinone core with ethanolamine present in membrane structures.

**Fig. 6:**
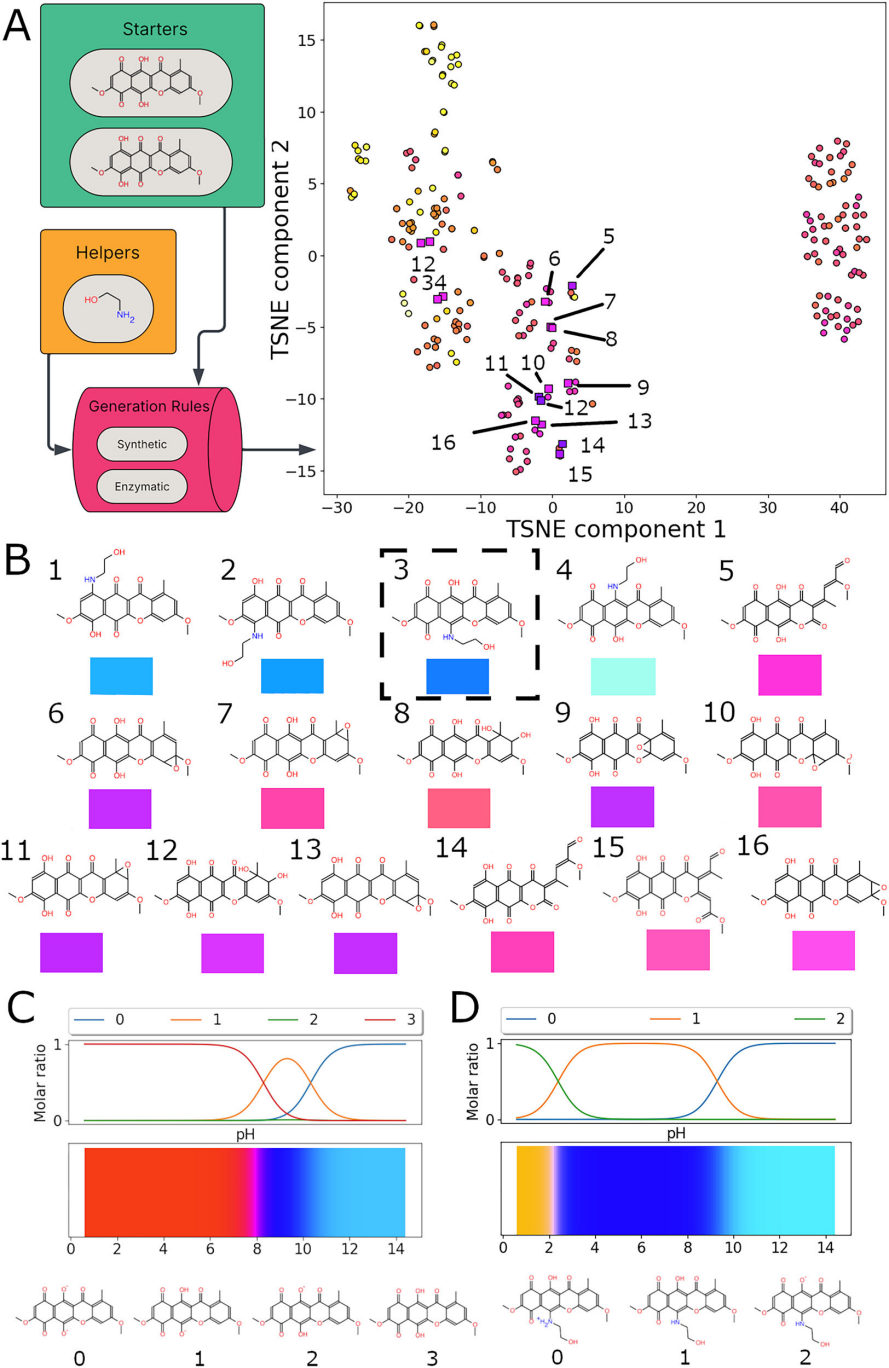
Bikaverin case study. **A** Schematic DORAnet workflow demonstrates the generation of bikaverin derivatives with predicted colour using the developed ML model. **B** A subset of bikaverin derivatives with the lowest calculated absorption energies, colour samples below each structure are based on quantum chemical calculations. The dashed-line box highlights the most thermodynamically stable ethanolamino-substituted bikaverin. **C**, **D**: colour vs. pH for bikaverin and its most stable amino derivative, respectively.

To explore potential reactions converting bikaverin to a blue product a pattern-based molecular generator DORAnet^43^ was applied and ethanolamine was added as a potential co-reactant. The MPNN-based colour prediction model was used to prefilter a set of generated structures and subsequent quantum chemical calculations (⍵B97X-D4 / def2-SVP (CPCM: MeOH) provided four bikaverin aminated derivatives predicted to be blue. Direct amination of naphthoquinones related to bikaverin proceeds smoothly with ammonia at room or slightly elevated temperatures^17^ confirming the possibility of the reaction with amino-containing substrates like proteins. Interestingly a blue colour was not predicted for any other generated compounds (Fig. 6). Halochromic properties were predicted for both bikaverin and the most stable aminated derivative. The transition from the red colour of the neutral form of bikaverin to the deprotonated blue species described in the literature at high pH is perfectly captured^16^. At the same time the aminated derivative is blue even at acidic pH which is usually used for cultivation of bikaverin natural producers.

## Conclusions

Microbial colourants like prodigiosin^44^ or actinorhodin^45^ are being explored for textile dyeing applications. They are produced in genetically engineered microorganisms and applied directly to the fabric as a cultural liquid. However in the case of these colourants, photostability is an issue. We have built the DyeDactic colour prediction workflow to guide synthetic biologists in the design of improved microbially produced colourants. The workflow starts with a computationally inexpensive ML approach to estimate electronic transition energies, which is applicable to the screening of large sets of potential colourants. This is followed by a TD-DFT approach applied to protonated and deprotonated molecular species. Depending on the calculated spectral properties (transition energies, oscillator strengths) and the pK_a_ values of colourants, colour as a function of pH was estimated and compared to experimental data for several well-known natural colourants. If necessary, the colour prediction methodology can be extended, employing more sophisticated and time-consuming techniques, such as including vibronic transitions with different levels of Franck–Condon factor estimation, or conformational sampling in the case of flexible molecules.

The collected data set is currently the largest resource of absorption spectra properties of natural colourants. A comparison of structural and physico-chemical properties of the data set and industrial colourants from the Colour Index leads to a set of suggestions to make coloured metabolites more suitable for industrial textile dyeing. Additionally, these spectral data helped to extend the scope of a published MPNN model trained on industrial colourants to biosynthetically accessible scaffolds. It decreased the MAE from 0.26 eV before fine-tuning to 0.18 eV, which is comparable to the MAE achieved by TD-DFT. Structural filters (size, asphericity, etc.) can be included in the filtering pipeline to guide the development of colourants belonging to a particular class of dyes.

Since natural colourants frequently demonstrate halochromic behaviour, we predicted colour based on the ionised species composition at different pH levels. In the case of rigid colourants with known composition, the colour predictions agree well with experimental data. However multiple sources of error, such as insufficient sampling in case of flexible molecules, pH induced chemical reactions, large pK_a_ prediction errors and aggregate formation at higher concentration, can lead to a dramatic difference between experiment and predictions. Some of these sources of error can be mitigated by conformational sampling with ions and explicit solvent present, with subsequent TD-DFT calculations for a representative set of structures. This approach gave a clear improvement in the case of biliverdin, which has significant conformational mobility. As a demonstration of the capabilities of the workflow, chemical derivatives of polyketide bikaverin achievable by one enzymatic or chemical reaction were explored and an explanation of the colour change was proposed. Working in conjunction with an appropriate structure generation engine, this multilevel approach will help to identify new biosynthetic colourants with properties required by the textile industry.

## Methods

### Data management and preprocessing

Three data sets were used in this work: a manually collected data set of natural colourants containing absorption spectra information; a subset of artificial dyes and pigments from colour index^46^, which contains information about colourant application but no spectroscopic data; and the data set of artificial dyes and pigments used by Greenman et al.^27^ to train their models derived from Deep4Chem^34^.

### Data set of natural colourants

A database of natural colourants was collected directly from the literature. It contains spectral information about 647 colourants from 413 sources and is provided in the project Github page^47^. All of the collected colourants have information at least about the highest absorption maxima and the solvent in which the spectral information was recorded. All natural colourants in the database were considered to be neutral without charged groups unless it was explicitly mentioned otherwise in the original research paper for a particular colourant, e.g. anthocyanines. If an absorption spectrum contains more than one peak in the UV-A and visible regions, then all relevant peaks were added to the database. Molar extinction coefficients were found for 375 colourants. One hundred seven colourants also have full spectra, which can be found in the corresponding publication. Each substance is characterised by a trivial name, a living organism species used for isolation and a SMILES string. Chemical space visualisation was carried out using the t-SNE^18^ technique by mapping RDkit circular fingerprint counts FCFP4^48^ (2048 bits) to a two-dimensional space.

### Dyes and pigments from Colour Index

Access to the Colour Index was purchased from SDC Enterprises Ltd. Colourants were downloaded using selenium (https://selenium-python.readthedocs.io/), yielding 4327 structures in JPG format. These files were converted to SMILES representations with the Decimer^49^ OCR engine and checked manually using JSME^50^. Inorganic pigments sulphur dyes and natural colourants were not considered further. Some colourants belonging to solvent acid and pigment classes, which exist as salts or metal complexes, were split into components and metals were removed leaving only the organic part for further consideration.

### Artificial colourants from Deep4Chem

A database of artificial dyes and pigments for fine-tuning the MPNN model was taken from the Zenodo page^51^ associated with the original MPNN transition energy prediction tool^27^. The data set was cleansed. Many data points had unrealistically large differences between calculated and mined experimental absorption maxima, which may have either been artefacts arising from text mining, or taken from transient absorption spectra. In any case data points from the artificial dyes with a wavelength difference larger than 100 nm between the experimental and calculated data after systematic correction were removed; this decreased the data set by 10%. Similar pruning was applied to the natural colourant data set also taking a threshold of 100 nm. This permissive threshold to detect outliers was adopted to retain as many natural colourants as possible. Random train-test splits were done for both natural and artificial colourant sets in a 9:1 proportion. The larger proportion was used as a single training set, while the rest of the artificial colourant data was used as a validation set in a hyperparameter optimisation procedure. The smaller part of the natural colourant database after the split was used as an external test set for estimating the accuracy of the final model. Workflow diagrams for data splits and ML training can be found in Schemes S1 and S2.

### MPNN training

Chemprop^26^ (v2.1.2) was used for the training and application of the MPNN models for the regression task of predicting transition energies which can potentially be any real number. To achieve this the very last layer before output of the neural network is linear. SMILES strings of colourants and solvents are the input data for the model, as in the original paper. The Hyperopt^52^ Python package was applied to fine-tune model hyperparameters: learning rate schedule, activation function, dropout probability, batch size, type of aggregation function, depths and sizes of neural networks used in the MPNN workflow. This package applies Bayesian optimisation to find a parameter set where an arbitrary function can reach minimal or maximal level. In our case the function was the mean squared error between the experimental and predicted transition energies on the validation set. Every run of the algorithm a neural network is instantiated and trained, performance assessed and the new parameter set is chosen for exploration based on performance on the previous algorithmic runs. The training process is organised in a sequence of so-called epochs and during each epoch each data point is shown to the model exactly once. Two hundred runs of the optimisation algorithm were performed and each model was trained for 30 epochs before estimating its performance. Finally a five-fold cross-validation procedure was applied to a model trained with the best parameter set identified during optimisation of hyperparameters. To create a train-validation-test split for each fold the following procedure was carried out. Artificial and natural colourant data sets are split into five subsets. One of the subsets of artificial colourants is used for validation and one of the natural colourant subsets serves as a test set. The rest of the data is joined to form a training set for the fold. An ensemble of four models was trained for each fold for 500 epochs. A transition energy prediction for a colourant is made using all 20 trained models and the average value is reported as a prediction result, while the standard deviation is calculated to estimate uncertainty.

### 3D structure generation procedure

The generation of a 3D structure for a TD-DFT calculation starts with a search for the most stable tautomer, followed by conformer generation and GFN2-xTB^28^ optimisation. To find the lowest energy tautomer, the following procedure is applied. First, the rdkit^43^ Python package is used to enumerate a maximum of 10,000 tautomers. Some tautomers with *sp*^2^-carbons in rings which are not present in the initial structure, are removed. For each tautomer three 3D conformations are generated in a high throughput mode using the EKTGv3^53^ approach implemented in rdkit^54^, minimised in MMFF94s^55^ and one conformation for each tautomer having the lowest energy is taken for further processing. Generation of at least ten conformations is preferable especially for flexible molecules. However there is frequently an enormous number of tautomers to check. So limiting the number of conformations to three is a reasonable compromise. Comparison of these two approaches is provided in the SI.

Next the relative energies of the tautomers are estimated using GFN2-xTB single-point energy calculations. The ALPB solvation model^56^ is applied using the solvent reported in the experimental paper, with all other parameters taken by default. If the reported solvent is not parameterised water is specified as the solvent. Finally ten conformations are generated for the lowest energy tautomer and fully optimised using GFN2-xTB with the same conditions for energy estimation. The lowest energy conformation is used for DFT and TD-DFT calculations.

All subsequent quantum chemical calculations are performed in ORCA 5.0.3^57^. Starting from the optimised conformation at the semiempirical level of theory an optimisation is performed with a composite PBEh-3c method^58^, which provides high-quality optimised geometries at low computational cost, with the CPCM^59^ model with the same solvent used for experimental measurements. Spectral properties for several colourants were measured in a solvent mix (gradient elution in HPLC, etc.); solvents with the highest fractions are specified for CPCM in these cases. Energy change thresholds of 10^−6^ and 5 × 10^−6^ Hartree are chosen for the SCF and geometry optimisation procedures, respectively. Resolution of identity for Coulomb integrals and chain of spheres approximation for exchange integrals (RIJCOSX^60^) is employed to speed up the SCF procedure. Default auxiliary basis sets are specified.

### TD-DFT calculations

The optimised structures are taken forward for the calculation of the electronic transition energies using TD-DFT. We explored a set of functionals recommended in the literature^61,62^ with different levels of sophistication: hybrid GGA (PBE0^63^, ⍵B97X-D4^32^, CAM-B3LYP^64^), hybrid meta-GGA (BMK^65^, M06-2×^66^) and several double hybrids (B2PLYP^67^, SCS-ωPBEPP86^31^, SCS-PBE-QIDH^31^]) with the application of CPCM solvation and the TDA^29^ (only with ⍵B97X-D4). A valence triple-zeta basis set^68^ (def2-TZVP) was used for neutral and cationic molecular species, while anions were treated with its augmented version^69^ (ma-def2-TZVP). Smaller basis sets def2-SVP and ma-def2-SVP were used for colour versus pH calculations for biliverdin, since the calculations with ma-def2-TZVP basis set converged slowly for negatively-charged biliverdin species. A stricter criterion was imposed on the wavefunction convergence (10^−8^ Hartree for energy change). Five roots were requested from the excited state calculations in general and ten roots to reproduce the full absorption spectrum of biliverdin. To compare with the literature and experimental data, only transitions with oscillator strengths above 0.01 were considered, i.e. we focused on ‘bright’ transitions.

A multilevel single-point TD-DFT calculation for trajectory frames from a replica exchange simulation^70^ was performed as follows only for biliverdin. This colourant was treated at the ⍵B97X-D4/ma-def2-SVP level without a solvation model and the TDA was not used. All molecules within 5 Å of the colourant were modelled using the GFN2-xTB model and all other molecules were treated at the MM level. Ten roots were requested to ensure full spectrum coverage of the UVA/vis range.

Vibronic effects were considered using the Frank–Condon vertical gradient approximation, which assumes adiabaticity of electronic transitions and employs the harmonic approximation for the nuclear motion. The excited state geometry is generated by following one optimisation step from the optimised ground state geometry using excited state gradients; the ground state Hessian matrix is used for the excited state as well. This consideration of vibronic effects was carried out for emodin, quinalizarin and orcein.

Transition energies obtained from TD-DFT demonstrate a systematic shift from the experimental positions of absorption maxima. To make a correction, a linear model with two parameters, slope and intercept, was trained. All calculated transition energies for colour prediction are corrected by application of this equation. The parameters for the tested functional are presented in Table S1.

### QM descriptors calculation and linear regression

All descriptors^37^ are calculated at the ⍵B97X-D4/def2-TZVP level of theory. The highest occupied molecular orbital (HOMO) and lowest unoccupied molecular orbital (LUMO) energies (in eV), as well as the absolute value of the dipole moments (μ, in Debye), are taken from the calculation directly. All other quantities including electronegativity (X), hardness (η), electrophilicity (ω), mean (α) and anisotropic (Δα) polarisabilities are calculated using equations (S1) to (S6) presented in the Supplementary Information. The calculated descriptor set is used to build linear regression models based on the Elastic Net approach^71^; details are provided in Supplementary Methods.

### Molecular dynamics simulations

Molecular dynamics (MD) simulations were performed for biliverdin in water. The General Amber force field^72^ and AM1-BCC^73^ charges were used to derive force field parameters, including partial charges for the colourants. The TIP3P water model together with the recommended ion parameters^74^, were applied to derive a parameter set for the system. The initial structure of the colourant (S11111 form, Scheme S8) was generated as described in Section 3D Structure Generation Procedure. The other seven forms were prepared by removing the required acidic hydrogen atoms manually. The systems for simulation were constructed using the Leap program^75^ from AmberTools 23 by placing the colourant molecules into an octahedral box where the shortest distance to the walls from any biliverdin atom is 12 Å. Water molecules and sodium and chloride ions were added to neutralise the system and keep the NaCl concentration at 0.1 M. All simulations were carried out using GROMACS 2020.3^76^. Simulation parameters can be found in the generation scripts in Github^47^. Replica exchange calculations include parallel simulation of a set of system instances at different temperatures. Higher temperatures provide a means for conformational changes which are slow at lower temperatures, but low temperatures allow one to obtain the correct ensemble of conformations for the experimental conditions. So in addition to integration of the equations of motion which is done in any MD simulation there are steps where geometries of replicas simulated at different temperatures are swapped with the probability calculated based on the potential energy difference between these two replicas of interest. Geometry optimisation of the system was performed and 26 replicas were generated and equilibrated for 1 ns at temperatures between 300 and 400 K with a 4 K step. Temperature steps of this size ensure an efficient swap of replicas during the subsequent 50 ns REMD simulation^68^. Ensembles of conformations produced at 300 K were extracted from trajectories after centring the colourant molecule in the centre of the box and used for multilevel TD-DFT calculations without any geometrical changes.

### Prediction of halochromic properties

For colour prediction as a function of pH the composition of protonated species is estimated and the following approximations are made to simplify the workflow. First no pH-induced chemical transformations happen except protonation and deprotonation; this condition is frequently satisfied. However there are examples of natural colourants, e.g., anthocyanins^77^, which do change their structure. Spectral changes due to colourant aggregation are not considered. Large molecules can disobey this rule, e.g., hypericin, in which absorption spectra at higher concentrations are shifted compared to those at lower concentrations^78^. No intermolecular interactions are taken into account except for bulk solvent effects as considered by the implicit solvation CPCM model, unless MD simulations are done. It is assumed that the absorption spectra are totally additive. Acidity microconstants are calculated using the ChemAxon pK_a_ prediction plugin in Chemaxon Playground^79^ and the distribution of microspecies is estimated as described in the literature^80^; equations are provided in SI. The populations of colourant microspecies were estimated by hardcoded functions (see the Github^47^ repository for more details) for each colourant.

The estimated populations of protonated and deprotonated species are used to choose significantly populated protonation states at the pH of interest, ranging from 1 to 13 which is relevant for textile dyeing, TD-DFT calculations are carried out and UV–vis absorption spectra are modelled. The spectral line shape in the case of a single conformation is approximated by a single Gaussian function with *σ* = 0.4 eV for simplicity and centred at the calculated transition energy after systematic error correction. In the case where the shape of the spectral line is modelled using the absorption of multiple conformations of the colourant, the absorption spectrum of each conformation is approximated by a Gaussian with *σ* = 0.08 eV centred on the corresponding transition energy after the systematic error removal (⍵B97X-D4/no CPCM), multiplied by the corresponding oscillator strength and the average value is calculated. The smaller value of *σ* is frequently used in the literature^81^ to model the spectral bandshape. All 5000 extracted frames from the simulations were used to ensure convergence. Slightly more frames with stronger broadening parameters were used than usually recommended^81^ as biliverdin is a flexible molecule. Then the modelled absorption spectra of species weighted by their populations at given pH are summed and converted to colour using the approach described in SI. In the case of line shape modelling using vibronic transitions the spectra calculated by ORCA for each transition were first convoluted with Gaussian function (*σ* = 0.08) for broadening, intensities scaled to be between 0 and 1 and multiplied by oscillator strength. Then all obtained spectra for a particular species of interest were added together and, finally, units of spectral intensities were converted to M^−1^ cm^−1^.

### Molecular generation example

A rule-based molecular generation engine, DORAnet^43^, was used to explore the local chemical space of bikaverin analogues. The two most stable tautomers of bikaverin were used as ‘starters’ and ethanolamine was used as a ‘helper’ molecule, due to its presence in natural blue polyketides. ‘helpers’ are structures which can potentially react with ‘starters’, but the generation of products from two ‘helpers’ is forbidden. In general, more helpers can be added, but we used only ethanolamine for illustrative purposes. Both synthetic and enzymatic rules were applied and 265 products were generated and shown in Fig. 6. Sixteen of the generated products with predicted absorption energies below 2.40 eV were passed to the next step of the workflow: TD-DFT calculations. The four structures predicted to be blue all result from phenolic hydroxyl substitution in bikaverin by the amino group of ethanolamine isomers. The colour dependence on pH was calculated for the most thermodynamically stable product (species 3 in Fig. 6).

### Experimental measurements of natural colourant absorption spectra

The following reagents are used in experiments: ethanol (LC-MS grade, ThermoFisher Scientific), DMSO (LC-MS grade, ThermoFisher Scientific), 2 M hydrochloric acid (Honeywell), anhydrous citric acid (>99%, ThermoFisher Scientific), glycine (BioUltra > 99.0%), K_2_HPO_4_ (>99%, Alfa Aesar), sodium hydroxide (98.5%, Acros Organics), quinalizarin (>95%, Merck), biliverdin (>97%, Merck), orcein (mixture, Merck), emodin (95%, BLDPharm). Buffer solutions for pH values ranging from 2 to 12 are prepared with the following chemicals: glycine (pH = 2.0, 9.0, 10.0), citric acid (pH = 3.0, 4.0, 5.0, 6.0) and K_2_HPO_4_ (pH = 7.0, 8.0, 11.0, 12.0). All buffer components are dissolved in deionized water (18.2 MΩ cm, Thermo Scientific Barnstead Smart2Pure) at 0.1 M concentrations and the required solution pH values are adjusted with 2 M NaOH or 2 M HCl solutions (Mettler Toledo FiveEasy pH-metre).

Five milligrams of each colourant is dissolved in 1 ml glycine buffer with pH 9 and 1 ml EtOH in case of anthraquinones or 1 ml of DMSO in case of orcein and biliverdin. Then the required amount of a colourant solution is added to each vial containing 2 ml of a buffer solution with 1 ml of EtOH to make the following concentrations of colourants (0.3 mM emodin, 0.1 M quinalizarin, 0.2 mM orcein (assuming the orcein is composed of only *α-*aminoorcein), 0.1 mM biliverdin). Photos of dissolved samples are obtained immediately before oxidation of alkaline solutions could take place. Then 200 μl of each sample is transferred to 96-well plates and absorption spectra are recorded (CLARIOstar Plus, BMG Labtech) together with spectra of control solutions. After subtraction of the background experimental spectra were used for colour assignment as described in the Supplementary Methods.

## Supplementary information

Supplementary Information

Description of Additional Supplementary Files

Supplementary Data 1

## Data Availability

The results from TD-DFT calculations and the trained chemprop models are available at https://github.com/colorifix/DyeDactic.

## Code availability

All code to reproduce the workflow (except the colour index related analysis) is available at https://github.com/colorifix/DyeDactic; 10.5281/zenodo.17955211.
