# Supplementary material for: DyeDactic workflow to predict halochromism of biosynthetic colourants
Source: Commun Chem. 2026 Jan 10;9:79. doi: 10.1038/s42004-025-01881-9 (PMC12894750; doi:10.1038/s42004-025-01881-9)
Supplement: Supplementary file 1 — Supplementary Information [file 42004_2025_1881_MOESM1_ESM.pdf]

Supplementary Information for "DyeDactic workflow to predict halochromism of biosynthetic colourants"

Dmitry S. Karlov[a,b], Rodolfo Marques[b], Richard J. Wheatley[a] and Jonathan D. Hirst[a]*

[a] School of Chemistry University of Nottingham, University Park, Nottingham, NG7 2RD, UK
[b] Colorifix Ltd, Innovation Centre, Norwich Research Park, NR4 7GJ

Table of contents

**Examples of tautomer generation and ranking**	**2**
**QM descriptors calculation**	**9**
**ElasticNet regression**	**9**
**Absorption spectrum to colour conversion**	**10**
**Generation of protonated species**	**10**
**General formula for absorption spectrum as a function of energy and pH**	**11**
**Scheme S1**	**12**
**Scheme S2**	**13**
**Scheme S3**	**13**
**Scheme S4**	**14**
**Scheme S5**	**14**
**Scheme S6**	**15**
**Scheme S7**	**15**
**Scheme S8**	**16**
**Figure S1**	**17**
**Figure S2**	**18**
**Table S1**	**18**
**Table S2**	**20**
**Table S3**	**21**
**Table S4**	**22**
**Table S5**	**24**
**Table S6**	**24**
**Table S7**	**25**
**Figure S3**	**26**
**Figure S4**	**27**
**Figure S5**	**27**
**Figure S6**	**28**

Table S8 28
Figure S7 30
Figure S8 31
Figure S9 32
Table S9 32
Figure S10 34
Table S10 35
References 37

Supplementary Methods

Examples of tautomer generation and ranking

To illustrate the applicability of tautomer generation and ranking approach four colourants (curcumin, resistomycin, prodigiosin, and bikaverin) were chosen and their conformation. Tautomers and the corresponding lowest energy conformations were generated and ranked with XTB energy of the corresponding conformation and the quality of ranking was assessed by comparison with *ab initio* method (PBE0/def2-SVP from PySCF[1]) for the same conformation. Relative energies in kcal/mol are provided. Both XTB and DFT approaches provide a very similar ranking of tautomeric species. The main discrepancies can be observed for high energy tautomers, but lowest energy tautomers are identical in all cases. Curcumin is known to exist as a mixture of keto and enol forms with the latter prevailing[2] and the correct ranking was captured by the algorithm. In case of bikaverin, the second highest ranked tautomer is present in X-ray structure[3] although energetic difference suggests that both forms can be observed at room temperature and stabilization of a particular tautomer can be facilitated by interactions with neighbours. Due to the probabilistic nature of the algorithm, conformations generated and optimised each time are slightly different which can affect the ranking order especially in case of tautomers with close electronic energy values. Nevertheless, both faster approaches (3 conformation per tautomer) and more thorough (10 conformation per tautomer) both provide the same most favourable tautomer in all cases.

Prodigiosin (10 conformations per tautomer)		
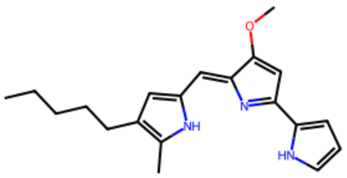 XTB: 0.0; PBE0/def2-svp: 0.0	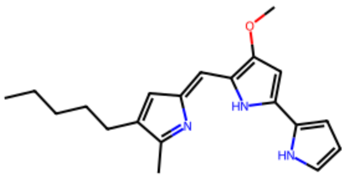 XTB: 6.6; PBE0/def2-svp: 9.9	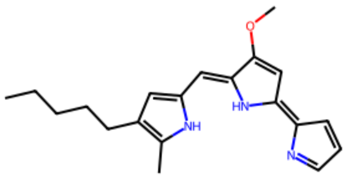 XTB: 14.8; PBE0/def2-svp: 21.1
Prodigiosin (3 conformations per tautomer)		
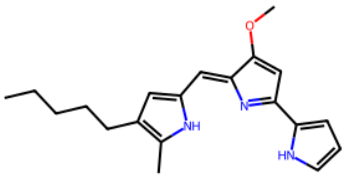 XTB: 0.0; PBE0/def2-svp: 0.0	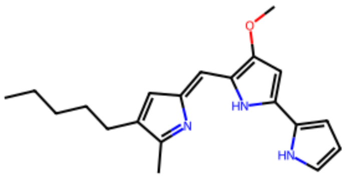 XTB: 2.2; PBE0/def2-svp: 2.6	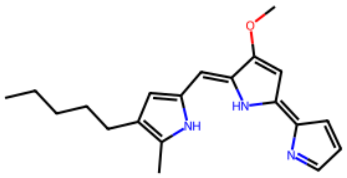 XTB: 12.4; PBE0/def2-svp: 15.2

Resistomycin (10 conformations per tautomer)		
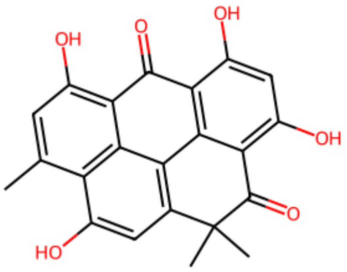 XTB: 0.0; PBE0/def2-svp: 0.0	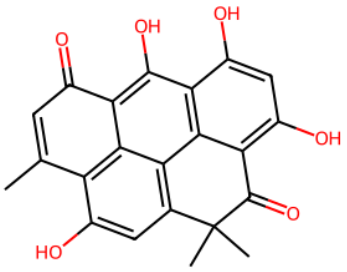 XTB: 5.8; PBE0/def2-svp: 7.6	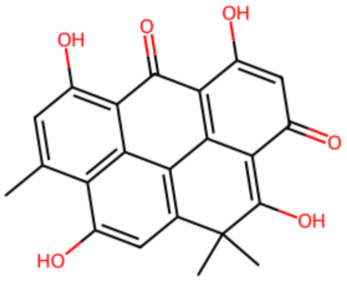 XTB: 12.3; PBE0/def2-svp: 16.3
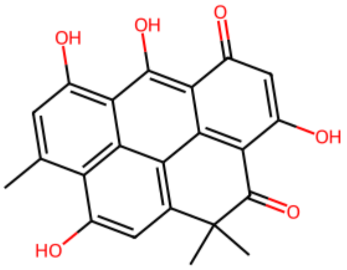 XTB: 14.3; PBE0/def2-svp: 19.9	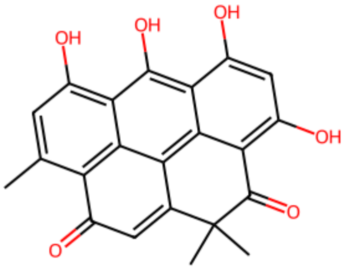 XTB: 15.1; PBE0/def2-svp: 24.8	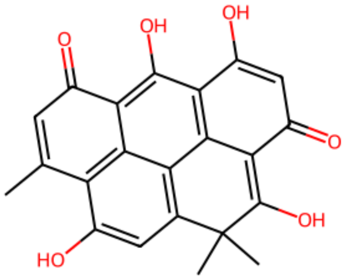 XTB: 16.2; PBE0/def2-svp: 20.8
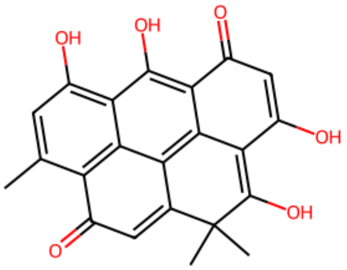 XTB: 21.4; PBE0/def2-svp: 28.5	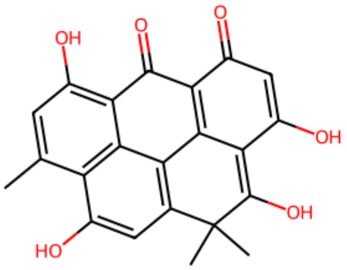 XTB: 22.2; PBE0/def2-svp: 39.8	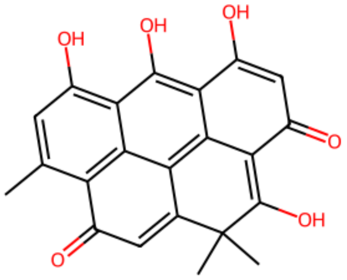 XTB: 22.2; PBE0/def2-svp: 30.3
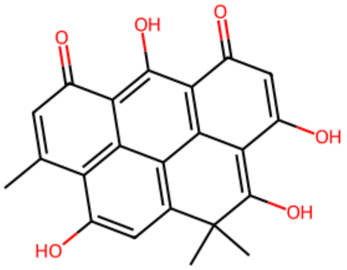 XTB: 23.4; PBE0/def2-svp: 40.4	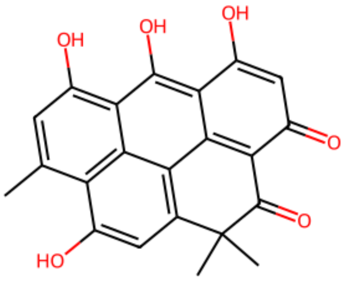 XTB: 24.3; PBE0/def2-svp: 44.0	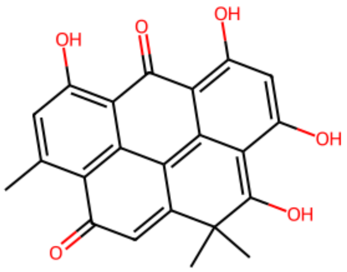 XTB: 24.8; PBE0/def2-svp: 33.3

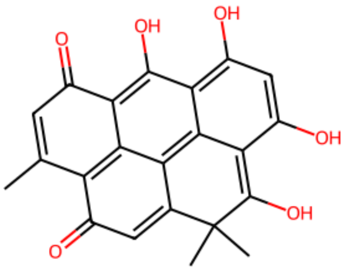 XTB: 38.5; PBE0/def2-svp: 51.0	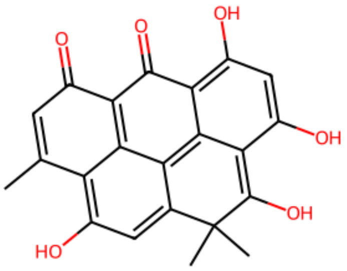 XTB: 39.6; PBE0/def2-svp: 64.9	
Resistomycin (3 conformations per tautomer)		
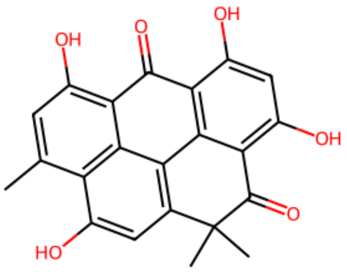 XTB: 0.0; PBE0/def2-svp: 0.0	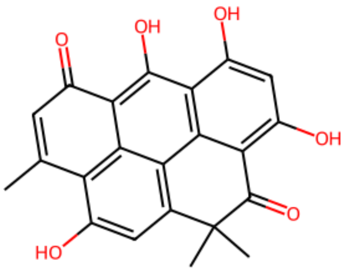 XTB: 5.8; PBE0/def2-svp: 7.6	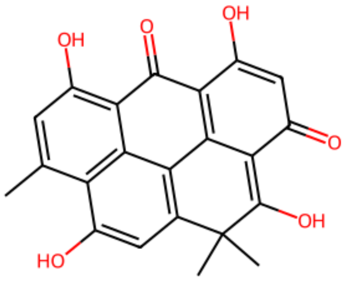 XTB: 12.3; PBE0/def2-svp: 16.4
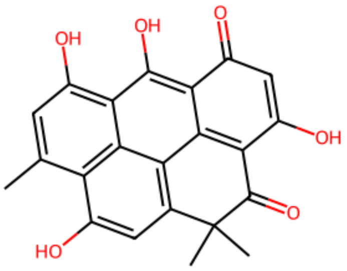 XTB: 14.3; PBE0/def2-svp: 20.0	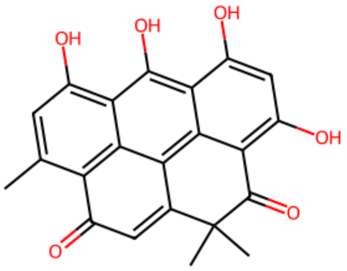 XTB: 15.2; PBE0/def2-svp: 24.9	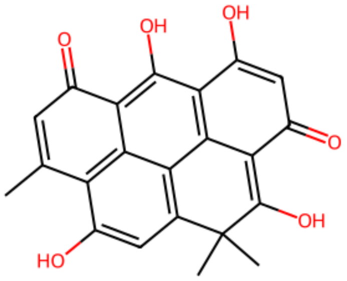 XTB: 16.2; PBE0/def2-svp: 20.8
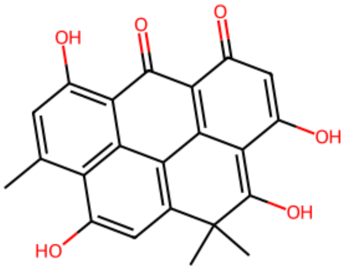 XTB: 22.2; PBE0/def2-svp: 39.9	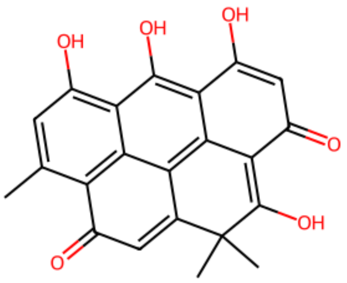 XTB: 22.2; PBE0/def2-svp: 30.4	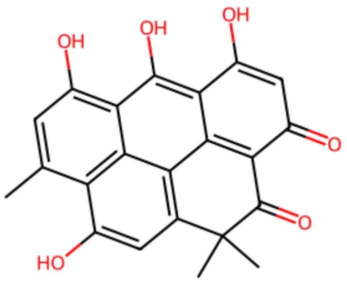 XTB: 24.3; PBE0/def2-svp: 44.1

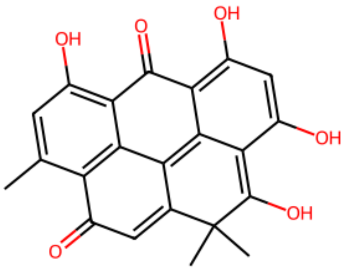

XTB: 24.8; PBE0/def2-svp: 33.4

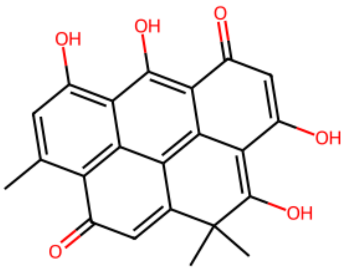

XTB: 25.1; PBE0/def2-svp: 39.3

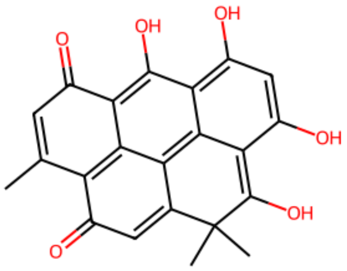

XTB: 38.5; PBE0/def2-svp: 51.1

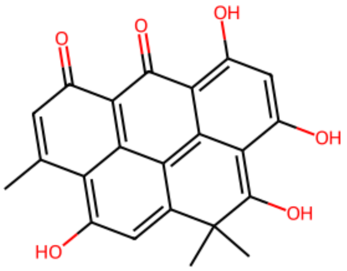

XTB: 45.6; PBE0/def2-svp: 70.6

Bikaverin (10 conformations per tautomer)

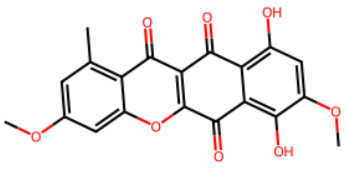

XTB: 0.0; PBE0/def2-svp: 0.0

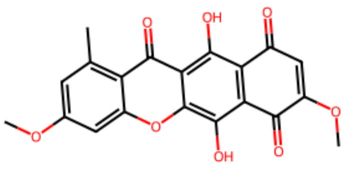

XTB: 0.9; PBE0/def2-svp: 0.3

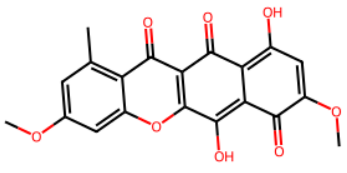

XTB: 6.2; PBE0/def2-svp: 8.3

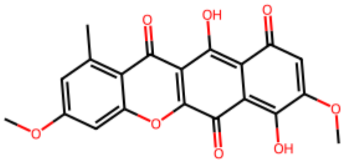

XTB: 11.4; PBE0/def2-svp: 18.4

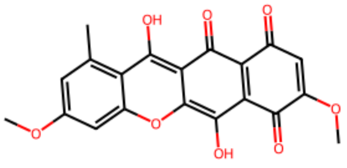

XTB: 17.0; PBE0/def2-svp: 23.2

Bikaverin (3 conformations per tautomer)

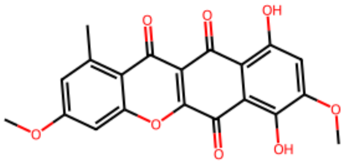

XTB: 0.0; PBE0/def2-svp: 0.0

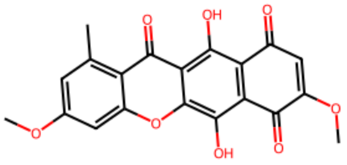

XTB: 4.0; PBE0/def2-svp: 2.6

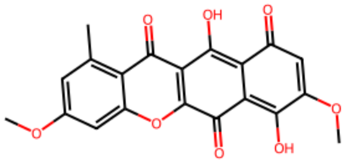

XTB: 8.7; PBE0/def2-svp: 10.7

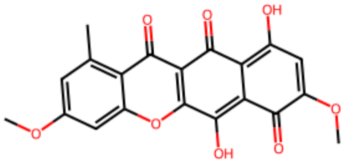

XTB: 9.1; PBE0/def2-svp: 9.9

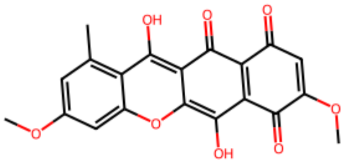

XTB: 20.7; PBE0/def2-svp: 26.3

Keto-curcumin (10 conformations per tautomer)

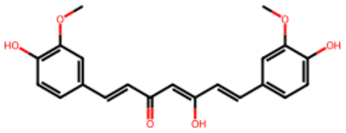

XTB: 0.0; PBE0/def2-svp: 0.0

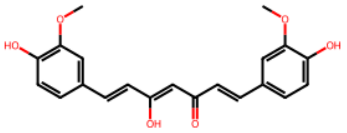

XTB: 0.3; PBE0/def2-svp: 2.9

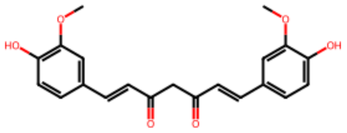

XTB: 1.6; PBE0/def2-svp: 3.4

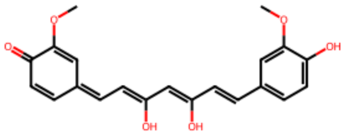

XTB: 17.9; PBE0/def2-svp: 22.3

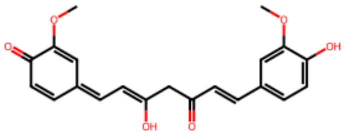

XTB: 19.3; PBE0/def2-svp: 19.7

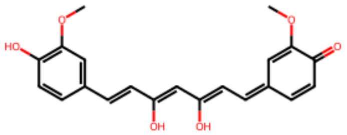

XTB: 19.5; PBE0/def2-svp: 24.4

<table>
<tr><td>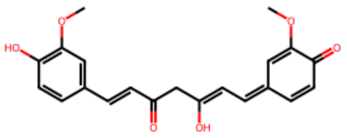
XTB: 20.2; PBE0/def2-svp: 23.5</td><td>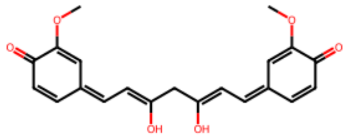
XTB: 37.1; PBE0/def2-svp: 45.4</td><td></td></tr>
<tr><td colspan="3">Keto-curcumin (3 conformations per tautomer)</td></tr>
<tr><td>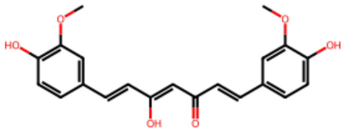
XTB: 0.0; PBE0/def2-svp: 0.0</td><td>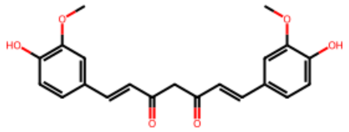
XTB: 1.9; PBE0/def2-svp: 3.3</td><td>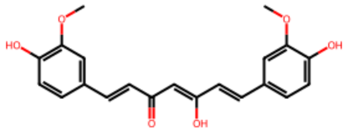
XTB: 5.3; PBE0/def2-svp: 14.8</td></tr>
<tr><td>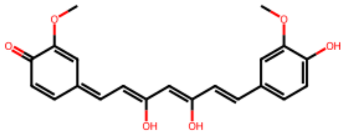
XTB: 21.2; PBE0/def2-svp: 33.2</td><td>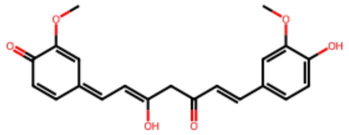
XTB: 21.8; PBE0/def2-svp: 23.9</td><td>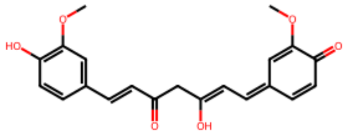
XTB: 22.1; PBE0/def2-svp: 28.7</td></tr>
<tr><td>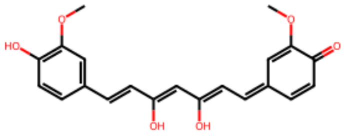
XTB: 22.7; PBE0/def2-svp: 31.3</td><td>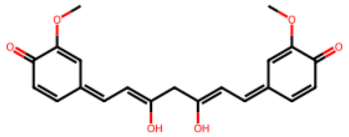
XTB: 37.9; PBE0/def2-svp: 47.4</td><td></td></tr>
<tr><td colspan="3">Enol-curcumin (10 conformations per tautomer)</td></tr>
</table>

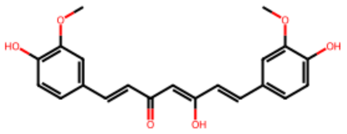<br>XTB: 0.0; PBE0/def2-svp: 0.0	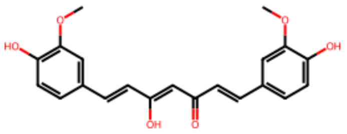<br>XTB: 5.4; PBE0/def2-svp: 14.8	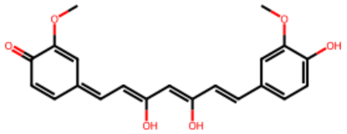<br>XTB: 18.7; PBE0/def2-svp: 25.8
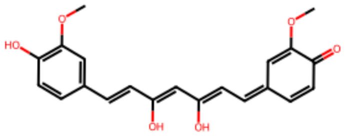<br>XTB: 23.1; PBE0/def2-svp: 33.9		
Enol-curcumin (3 conformations per tautomer)		
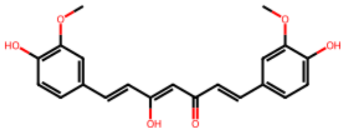<br>XTB: 0.0; PBE0/def2-svp: 0.0	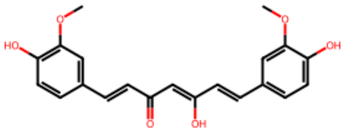<br>XTB: 4.8; PBE0/def2-svp: 8.4	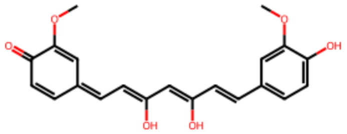<br>XTB: 21.8; PBE0/def2-svp: 31.8
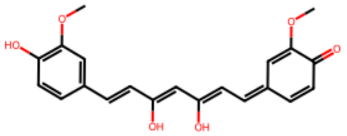<br>XTB: 22.5; PBE0/def2-svp: 34.2		

QM descriptors calculation

Dipole moment (μ):

$$\mu = \sqrt{\mu_x^2 + \mu_y^2 + \mu_z^2} \text{ (Eqn S1)}$$

Electronegativity (χ):

$$\chi = \frac{-(HOMO + LUMO)}{2} \text{ (Eqn S2)}$$

Hardness (η):

$$\eta = LUMO - HOMO \text{ (Eqn S3)}$$

Electrophilicity (ω):

$$\omega = \frac{\chi^2}{2\eta} \text{ (4)}$$

Mean polarizability (α) and anisotropic polarisability (Δα) were taken from polarizability tensor below computation of which was computed separately in ORCA by initiating CP-SCF procedure and left as they were in a.u.

Mean polarisability is

$$\alpha = \frac{\alpha_{xx} + \alpha_{yy} + \alpha_{zz}}{3} \text{ (Eqn S5)}$$

Anisotropic polarisability is

$$\Delta\alpha = \sqrt{\frac{(\alpha_{xx} - \alpha_{yy})^2 + (\alpha_{xx} - \alpha_{zz})^2 + (\alpha_{yy} - \alpha_{zz})^2 + 6(\alpha_{xy}^2 + \alpha_{xz}^2 + \alpha_{yz}^2)}{2}} \text{ (Eqn S6)}$$

ElasticNet regression

The idea behind this is to modify regression loss to tackle the problem of correlated features and select a subset of features. The predicted value of the *i*'th observable $\hat{y}_i$, is defined in equation (Eqn. S7). There are *N* data points and *M* is the length of a descriptor vector. y_i, θ_j, x_{ij}, are the observed value of the *i* dependent variable, *j* coefficient in the regression equation (7), and the value of *j* descriptor for *i* observation.

$$\hat{y}_i = \theta_0 + \theta_1 x_{i1} + \ldots + \theta_M x_{iM} \text{ (Eqn S7)}$$

In ordinary linear regression, the loss function used for optimisation is MSE_{loss} (Eqn. S8):

$$MSE_{loss} = \sum_{i=1}^{N} (y_i - \hat{y}_i)^2 \text{ (Eqn S8)}$$

In the modified loss function $ElasticNet_{loss}$ (Eqn. S9), which includes the error minimization term as well,

$$ElasticNet_{loss} = MSE_{loss} + \alpha(\gamma L_1 + (1-\gamma)L_2) \text{ (Eqn S9)}$$

There are two additional terms (L_1 (Eqn. S10) and L_2 (Eqn. S11)) to keep the regression coefficients closer to zero and prevent overfitting.

$$L_1 = \sum_{j=1}^{M} |\theta_j| \text{ (Eqn S10)}$$

$$L_2 = \sum_{j=1}^{M} \theta_j^2 \text{ (Eqn S11)}$$

The optimal model is selected by variation of coefficients α (loss magnitude) and γ (weight of each loss type). Both losses have their own advantages: L_2 frequently gives slightly better

models in terms of error, while L_1 loss drives highly correlated coefficients to zero. Ten-fold cross-validation with random splitting was done to find the optimal α and γ.

Absorption spectrum to colour conversion

A Python script was prepared to convert an arbitrary absorption spectrum into a colour. Absorption (either in optical density (OD) or transmission form) is applied to a spectrum of a standard D65 illuminant[4] (D65(λ)) decreasing the light intensity in certain spectral regions yielding light $I(\lambda)$ - (Eqn. S12). The resulting spectrum is numerically integrated with CIE XYZ colour matching functions $x_0(\lambda)$, $y_0(\lambda)$, $z_0(\lambda)$ (Eqns S13 to S15) calculating *X*, *Y*, *Z* tri-stimulus values divided by *N* (Eqn. S16). Integration is done for a range of wavelengths 380 to 780 nm. Each of *X*, *Y*, *Z* values were normalised by a sum of all of them. Finally, the XYZ vector can be converted to RGB values by a linear transformation **M** (a standard 3 x 3 matrix). Adobe RGB (1998) with D65 reference white was chosen in this work[5]. A gamma correction was not applied and the RGB vector was used to visualise colours.

$$I(\lambda) = 10^{-OD(\lambda)} \times D65(\lambda) \text{ (Eqn S12)}$$

$$X = \frac{1}{N}\int_{\lambda_{min}}^{\lambda_{max}} x_0(\lambda)I(\lambda)d\lambda \text{ (Eqn S13)}$$

$$Y = \frac{1}{N}\int_{\lambda_{min}}^{\lambda_{max}} y_0(\lambda)I(\lambda)d\lambda \text{ (Eqn S14)}$$

$$Z = \frac{1}{N}\int_{\lambda_{min}}^{\lambda_{max}} z_0(\lambda)I(\lambda)d\lambda \text{ (Eqn S15)}$$

$$N = \int_{\lambda_{min}}^{\lambda_{max}} y_0(\lambda)D65(\lambda)d\lambda \text{ (Eqn S16)}$$

Generation of protonated species

A compound with *N* ionisable groups can potentially exist in 2^N protonated forms since, generally, there is no structural symmetry and protonation centers are distinguishable from each other. For example, if we consider emodin which has 3 protonation centers (Scheme 2), let's then enumerate all protonation centers and assign species identifiers according to the following convention. For example, "S101" is a protonation state of emodin where the first and the third acidic groups are protonated while the second hydroxyl group is not. And the equilibrium constant between arbitrary species described by Reaction 1 will be defined in general by the equation (Eqn. S17).

$$SXYZ \leftrightarrow SPQR + (X + Y + Z - P - Q - R)\, H^+ \text{ (Reaction 1)}$$

$$K_{PQR}^{XYZ} = \frac{[SPQR][H^+]^{X+Y+Z-P-Q-R}}{[SXYZ]} \text{ (Eqn. S17)}$$

2^N-1 equilibria are enough to define concentrations of all species and this approach can be expanded to derive the equilibria for compounds with an arbitrary number of protonation centers. But if species with low populations are omitted then the number of equilibria can be much smaller.

Using Eqn. S17 we can derive concentrations of any microspecies [SXYZ] using the concentration of fully deprotonated form [S000] (Eqn. S18). X, Y, Z are either zero or one.

$$[SXYZ] = \frac{[S000]\,[H^+]^{X+Y+Z}}{K_{000}^{X00}\, K_{X00}^{XY0}\, K_{XY0}^{XYZ}} \text{ (Eqn. S18)}$$

Consider different paths through the graph from fully deprotonated form S000 leading to the partially protonated compound SXYZ (Eqn. S19). Since the concentration of the fully deprotonated form and hydronium ions are constant we can write the following equations:

$$K_{000}^{Y00}\,K_{0Y0}^{XY0}\,K_{XY0}^{XYZ} = K_{000}^{X00}\,K_{X00}^{XY0}\,K_{XY0}^{XYZ} = \ldots = K_{000}^{00Z}\,K_{00Z}^{0YZ}\,K_{0YZ}^{XYZ} = K_{000}^{X00}\,K_{X00}^{X0Z}\,K_{X0Z}^{XYZ} \quad \text{(Eqn. S19)}$$

Assuming each of the acidic sites has independent pKa we can write the following solution to (Eqn. S20).

$$K_{000}^{X00} = K_{0Y0}^{XY0} = K_{0YZ}^{XYZ} = K_1 \text{ and } K_{000}^{0Y0} = K_{X00}^{XY0} = K_{X0Z}^{XYZ} = K_2 \text{ and } K_{000}^{00Z} = K_{X00}^{X0Z} = K_{XY}^{XYZ} = K_3 \quad \text{(Eqn. S20)}$$

All these constants written above are calculated using ChemAxon pKa predictor. Given this approximation we can write the molar fraction for any microspecies of emodin (Eqn. S21):

$$x_{XYZ} = \frac{[H^+]^{X+Y+Z}/(K_{000}^{X00}\,K_{X00}^{XY0}\,K_{XY0}^{XYZ})}{1 + [H^+]/K_1 + [H^+]/K_2 + [H^+]/K_3 + [H^+]^2/(K_1\,K_2) + [H^+]^2/(K_1\,K_3) + [H^+]^2/(K_2\,K_3) + [H^+]^3/(K_1\,K_2\,K_3)} \quad \text{(Eqn. S21)}$$

The above approach can be extended to any compound which has protonatable atoms. At any given pH the calculated molar fractions are used to weight absorption spectra of all microspecies. The subsequent addition of them provides an absorption spectrum of equilibrium mixture as a function of pH.

General formula for absorption spectrum as a function of energy and pH

Let's put everything together. Since we now have pH-dependent molar fractions for protonation species defined in the previous section. The only thing left is to convert an oscillator strength (f_{osc}) to the corresponding extinction coefficient (ε). To do there is an equation for spectral intensity as a function of energy when absorption band is modelled by a Gaussian function (Eqn. S22)[6] which connects oscillator strength to the simulated absorption spectrum in $(M \times cm)^{-1}$. N_A is an Avogadro number, *c* - speed of light, *s* - standard deviation for a Gaussian function, E_i and f_c^{osc} are the *i* electron transition and oscillator strength, respectively.

$$OD(E) = \frac{\pi N_A}{3\sqrt{2\pi}\, ln(10)\hbar\varepsilon_0 c\, s}\sum_{i=1}^{N} f_i^{osc} e^{-0.5(\frac{E-E_i}{s})^2} = \frac{11451.73}{s}\sum_{i=1}^{N} f_i^{osc} e^{-0.5(\frac{E-E_i}{s})^2} \quad \text{(Eqn. S22)}$$

Taking into account the precalculated pH-dependent molar fractions (x_j - molar fraction for j species), total concentration of colourant (C_{col}) and the length of optical path (*l* = 1 cm) we can write the final equation (Eqn. S23). *M* and *N* are numbers of protonated species and electron transitions in visible range for a *j* protonated form.

$$OD(E,pH) = \frac{11451.73 * C_{col} * l}{s}\sum_{j=1}^{M} x_j(pH)\sum_{i=1}^{N} f_i^{osc} e^{-0.5(\frac{E-E_i}{s})^2} \quad \text{(Eqn. S23)}$$

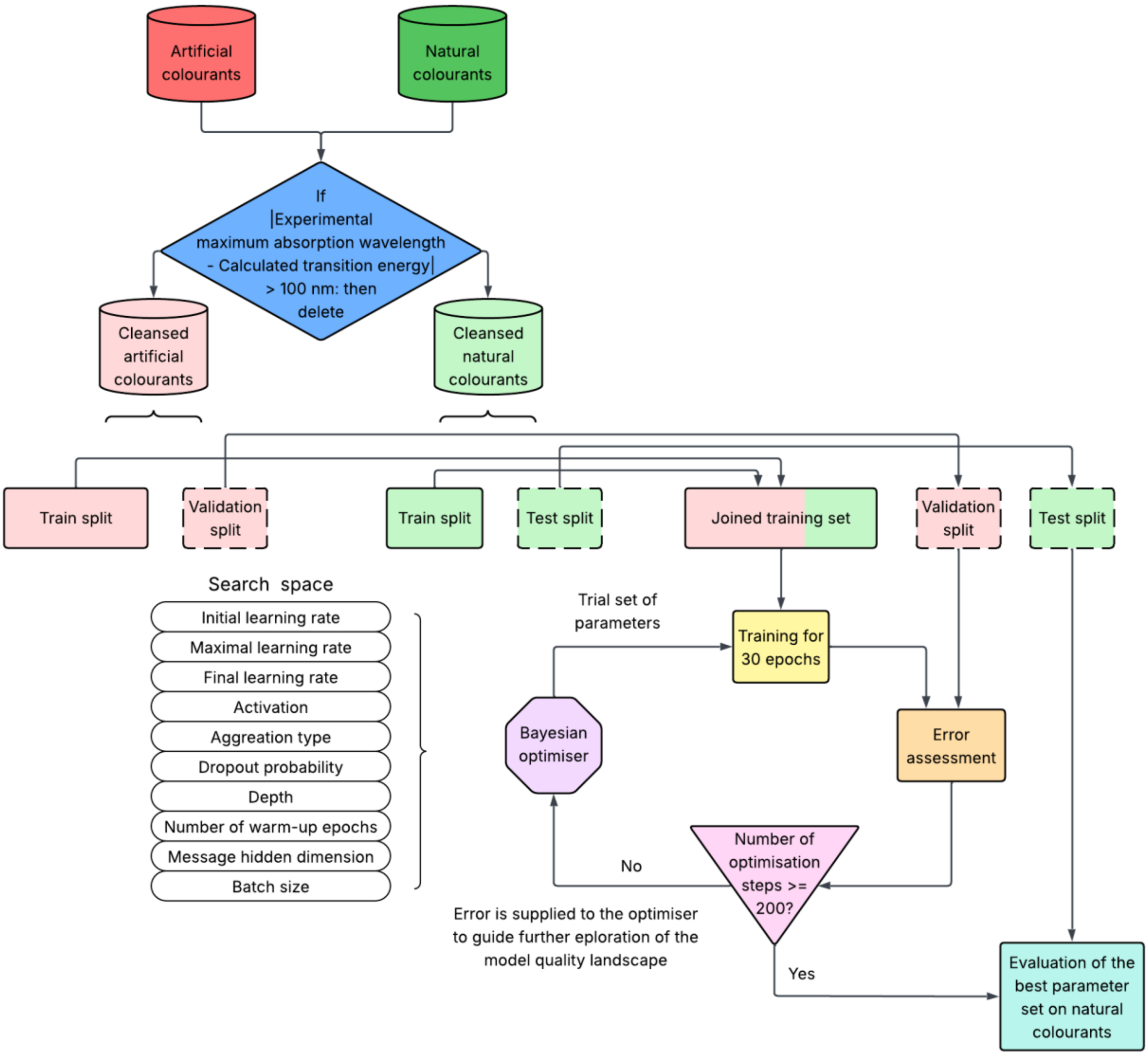

Scheme S1. Data splitting for neural network hyperparameter optimisation with a predefined train-test split. Chart is made in Lucidchart (https://www.lucidchart.com/)

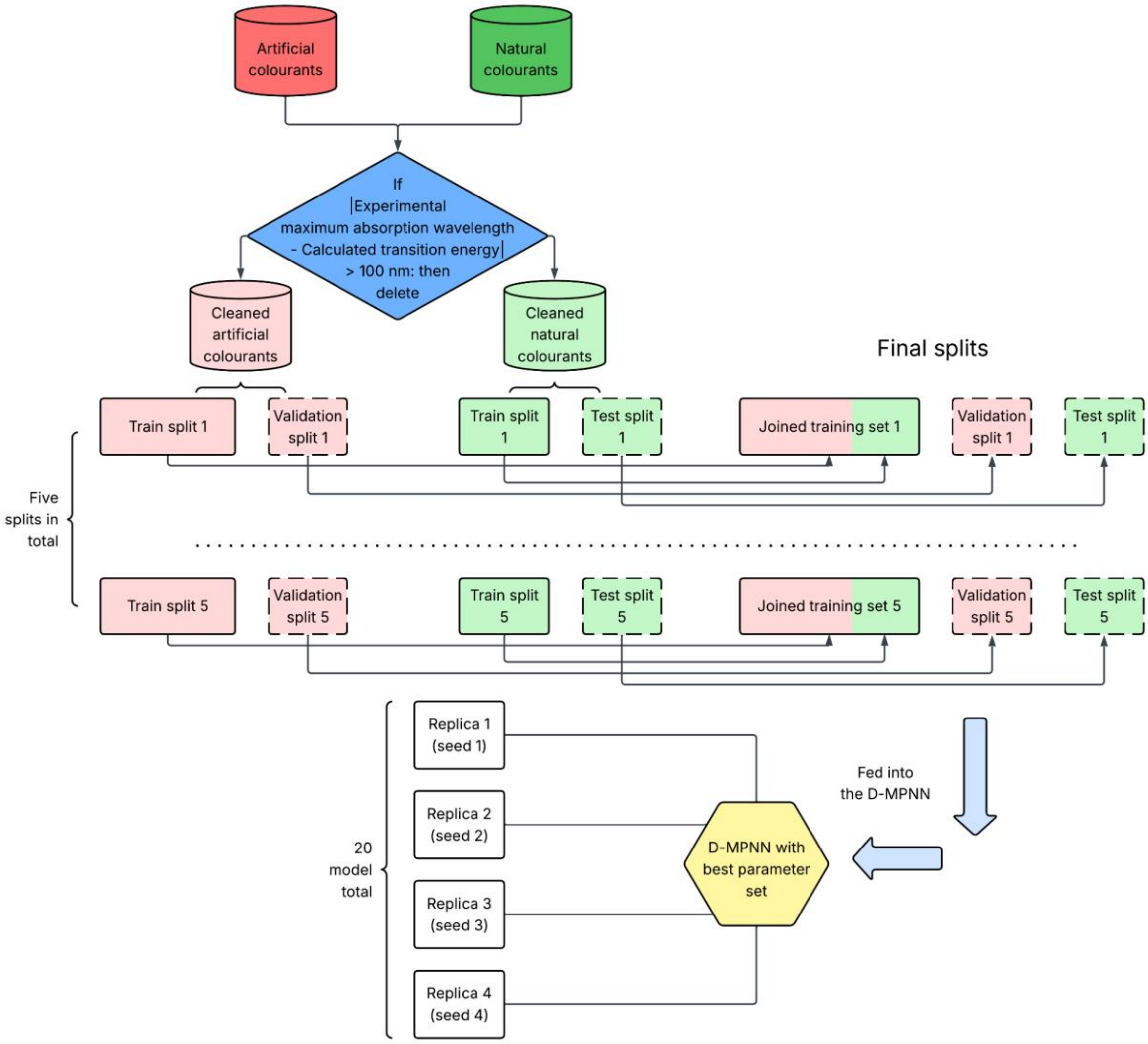

Scheme S2. Data splitting scheme for final neural network training with 5-fold cross-validation. Chart is made in Lucidchart (https://www.lucidchart.com/)

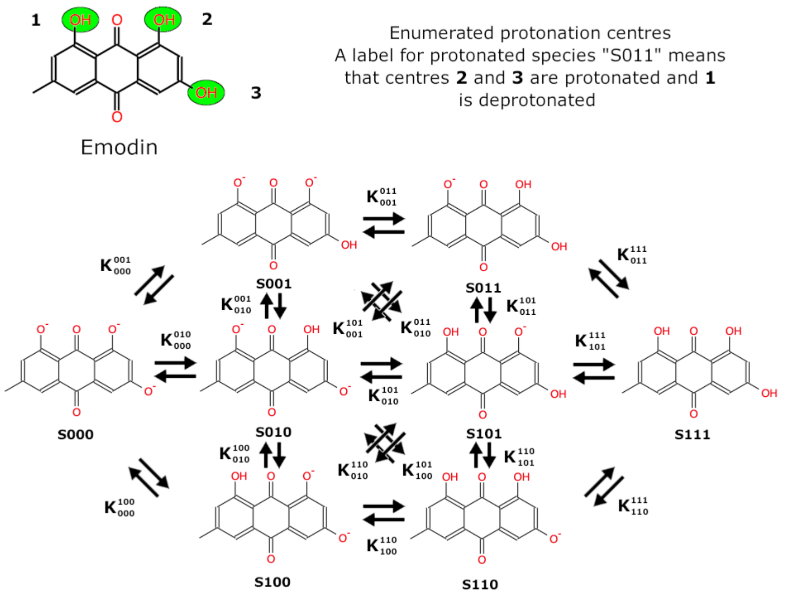

Scheme S3. A microstate equilibrium for emodin. Equilibrium like **S001** ⇄ **S110** or **S000** ⇄ **S011** are not shown to keep the Scheme clear but they are still implied.

Certain species can have negligible molar ratios at any given pH (< 0.01), so for the sake of saving computational time TD-DFT calculations were not carried out for these protonatable and not taken into account in the ionisation graph. Ionisation graphs for emodin, quinalizarin, α-hydroxyorcein, α-aminoxyorcein, and biliverdin are defined below.

Emodin:

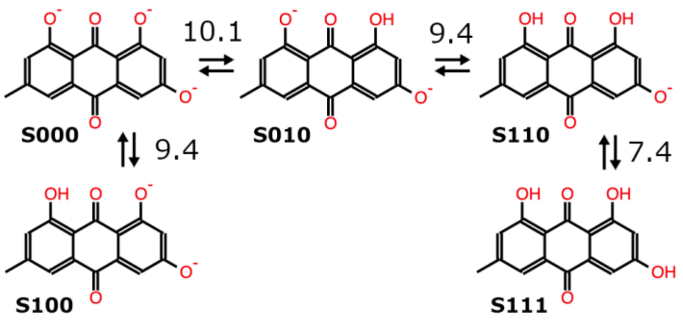

Scheme S4. Ionisation graph for emodin. Species with low populations are not included (<0.01 at any given pH). Acidity constants (pK_a) for corresponding protonation events are written above the equilibrium arrows. Numeration of protonation centers is provided in Scheme 2 and the predicted acidity constants are taken from Figure S26.

Quinalizarin:

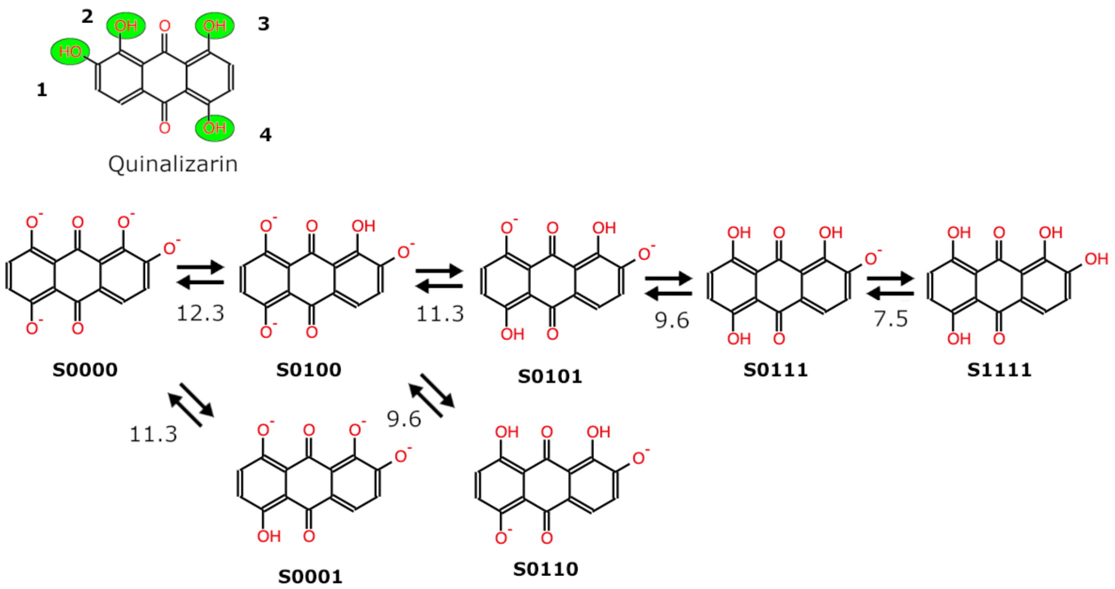

Scheme S5. Ionisation graph for quinalizarin. Species with low populations are not included (<0.01 at any given pH). Acidity constants (pK_a) for the corresponding protonation events are written below the equilibrium arrows. The predicted acidity constants are shown in Figure S26. Numbers on the top molecule mark the position of where the protonation state is encoded in a binary format.

α-hydroxyorcein:

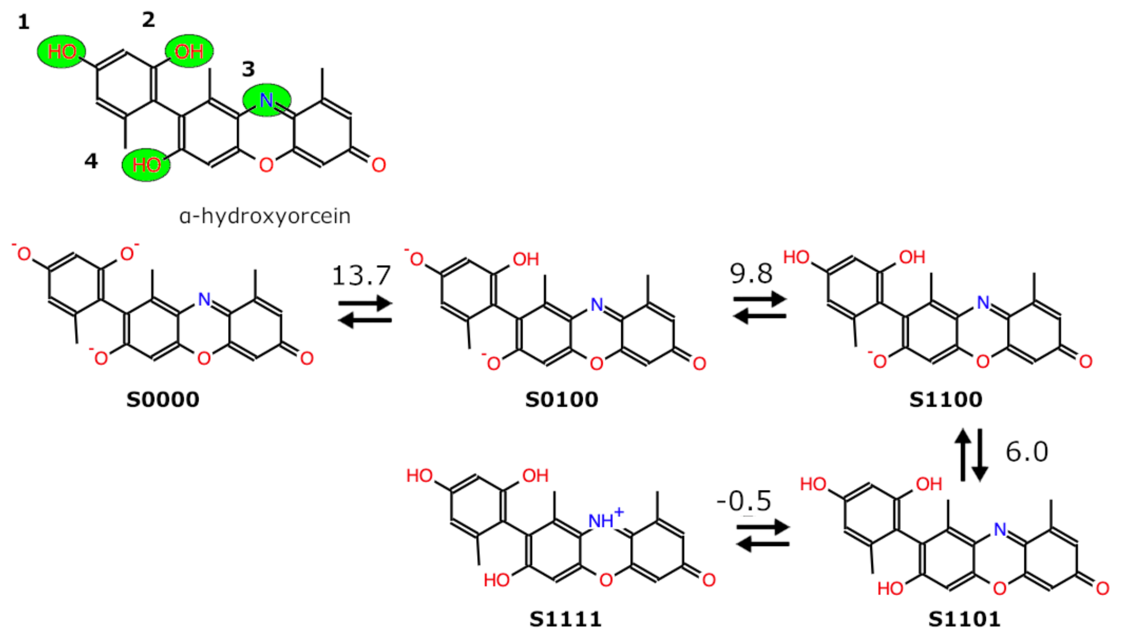

Scheme S6. Ionisation graph for α-hydroxyorcein. Species with low populations are not included (<0.01 at any given pH). Predicted acidity constants (pK_a) for the corresponding protonation events are written above the equilibrium arrows. The predicted acidity constants are shown in Figure S26. Numbers on the top molecule mark the position of where the protonation state is encoded in a binary format.

α-aminoorcein:

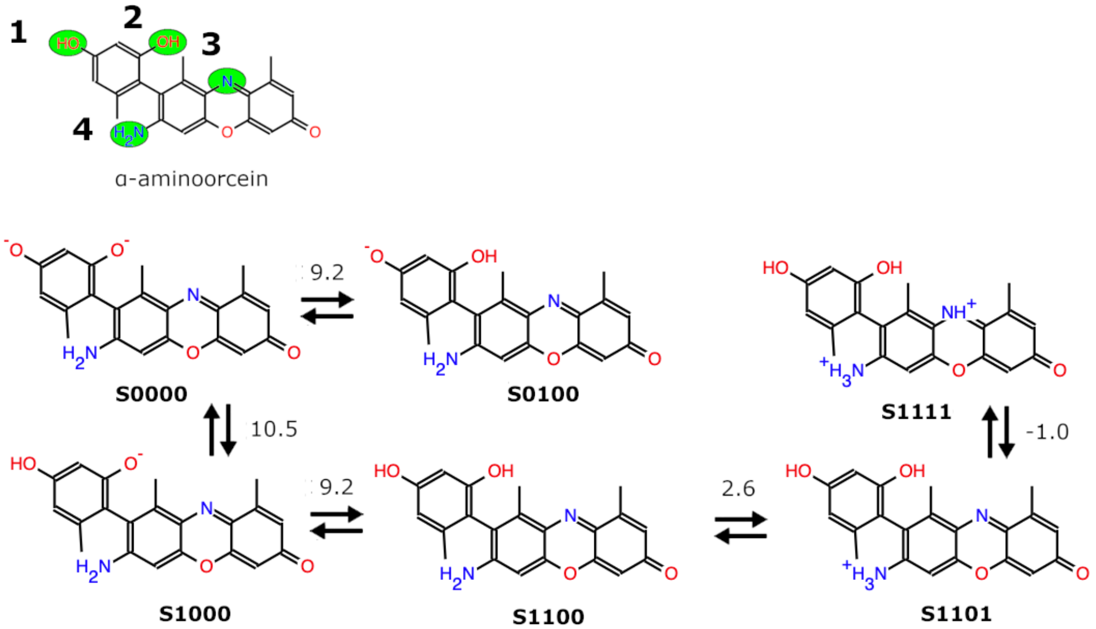

Scheme S7. Ionisation graph for α-aminoorcein. Species with low populations are not included. Predicted acidity constants (pK_a) for the corresponding protonation events are written above the equilibrium arrows. The predicted acidity constants are shown in Figure S26. Numbers on the top molecule mark the position of where the protonation state is encoded in a binary format.

Biliverdin:

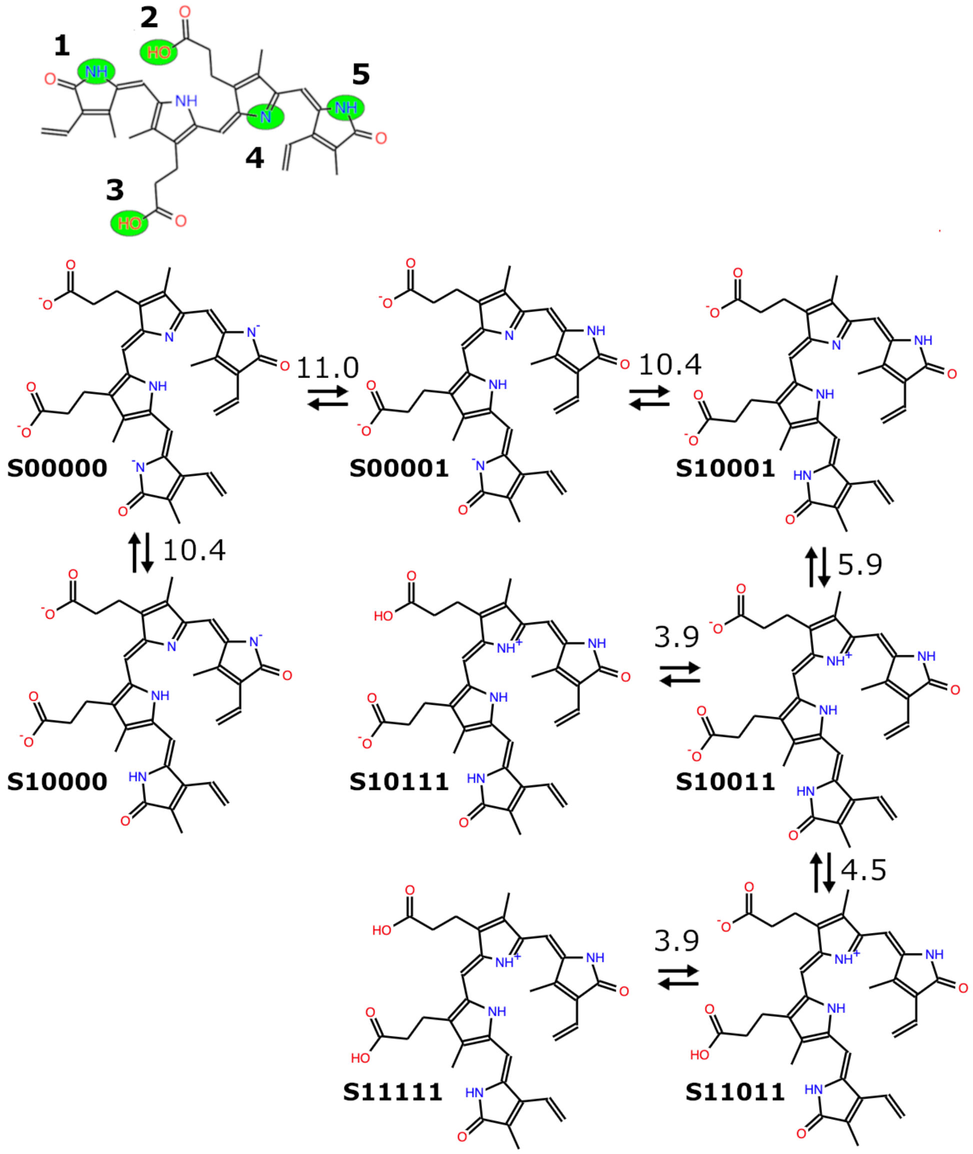

Scheme S8. Ionisation graph for biliverdin. Species with low populations are not included. Predicted acidity constants (pK_a) for the corresponding protonation events are written above the equilibrium arrows. The predicted acidity constants are shown in Figure S26. Numbers on the top molecule mark the position of where the protonation state is encoded in a binary format.

Supplementary Discussion

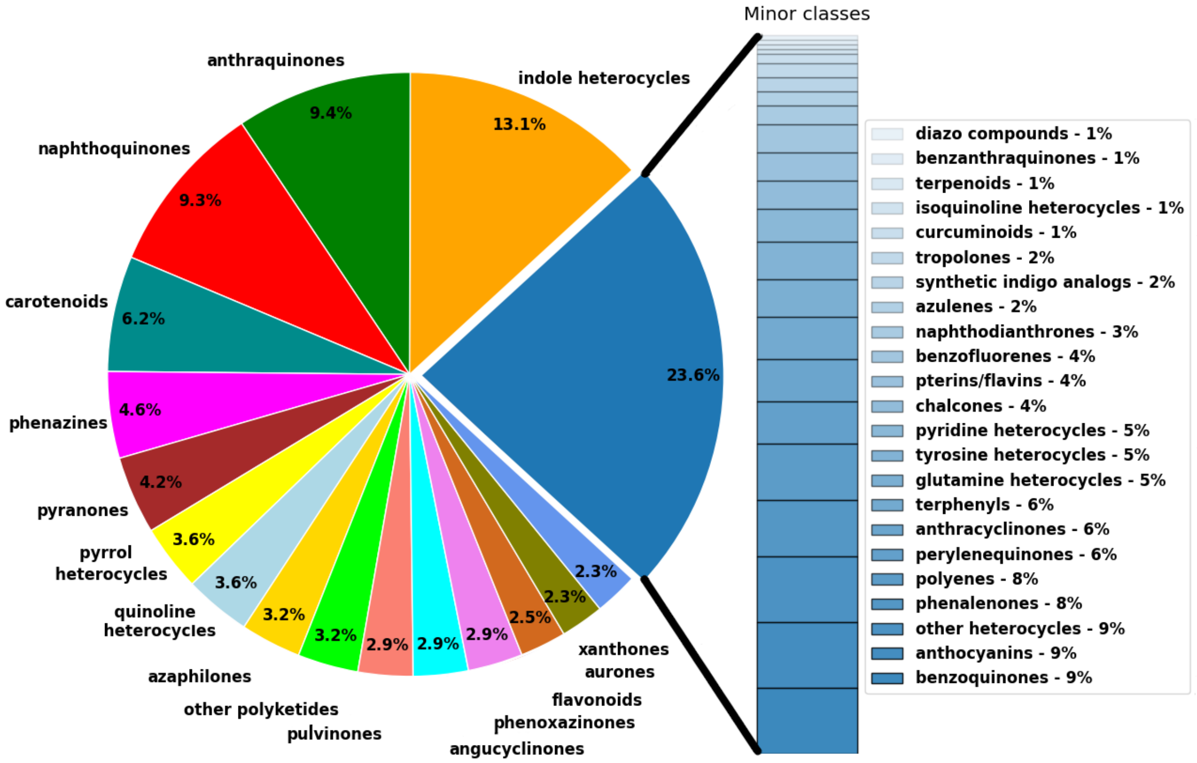

Figure S1. A pie diagram illustrating classes of natural colourants reported in this work. Colourant classes in the "pie" are marked with their fraction in the whole database while the percentage values shown for minor classes are taken from the fraction of the database (blue) which does not include more populated classes.

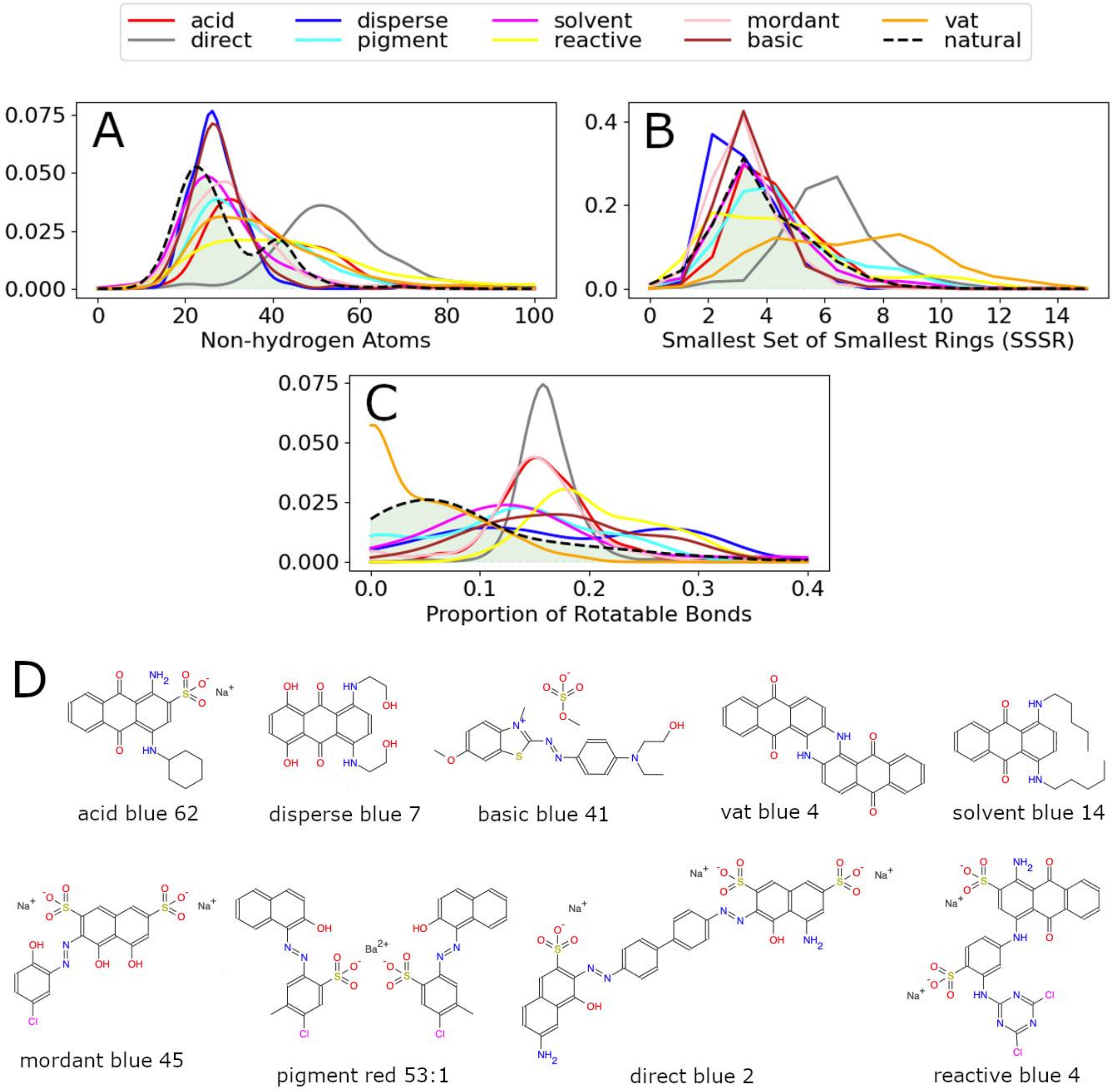

Figure S2. A comparison of distributions of colourants' simple structural properties: (A) non-hydrogen atoms, (B) number of rings, (C) proportion of rotatable bonds in overall number of bonds. A colour of the line marks the class of industrial dye while a black dashed line with green area under the line is used for the natural colourant database, and (D) examples of artificial dyes belonging to each class.

Table S1. Coefficients of linear regression used to eliminate systematic error of a TD-DFT calculation based on relationships between experimental absorption energies and calculated vertical transition energies. Solvent medium modelled by CPCM solvation is the same solvent used for experimental measurements.

$E_{corrected} = E_{calc} * slope + intercept$		
Method	Slope	Intercept, eV
PBE0	0.678	0.860
PBE0 + CPCM	0.678	0.920

ωB97X-D4	0.722	0.346
ωB97X-D4 + CPCM	0.720	0.432
ωB97X-D4 + TDA	0.719	0.232
ωB97X-D4 + TDA + CPCM	0.718	0.320
BMK	0.694	0.605
BMK + CPCM	0.690	0.684
CAM-B3LYP	0.720	0.597
CAM-B3LYP + CPCM	0.718	0.669
M06-2X	0.715	0.500
M06-2X + CPCM	0.714	0.573
B2PLYP	0.712	0.731
B2PLYP + CPCM	0.750	0.686
SCS-PBE-QIDH	0.725	0.572
SCS-PBE-QIDH + CPCM	0.773	0.504
SCS-ωPBEPP86	0.712	0.652
SCS-ωPBEPP86 + CPCM	0.748	0.629

Table S2. In-class Pearson correlation coefficients between experimental energies of the most intensive light absorption and vertical transition energies obtained by different methods for the natural pigments database. Green colour marks significant correlation (P-value < 0.05). This table contains classes of colourants with more than 5 members.

Class	PBE0	PBE0 solv	ωB97XD4	ωB97XD4 solv	BMK	BMK solv	CAMB3LYP	CAMB3LYP solv	M06-2X	M06-2X solv	B2PLYP	B2PLYP solv	PBEQIHD	PBEQIHD solv	ωPBEPP86	ωPBEPP86 solv
angucyclinones	0.14	0.31	0.34	0.49	0.25	0.40	0.21	0.38	0.36	0.45	0.24	0.42	0.38	0.55	0.44	0.60
anthocyanins	0.52	0.67	0.57	0.49	0.61	0.74	0.58	0.70	0.70	0.73	0.44	0.71	0.54	0.66	0.61	0.62
anthraquinones	0.88	0.85	0.90	0.88	0.89	0.86	0.89	0.87	0.89	0.87	0.90	0.88	0.91	0.90	0.91	0.90
aurones	-0.0	0.22	-0.0	0.14	0.03	0.21	0.05	0.23	0.03	0.22	-0.0	0.22	-0.1	0.20	-0.1	0.18
azaphilones	0.84	0.87	0.89	0.91	0.87	0.90	0.86	0.90	0.88	0.91	0.85	0.90	0.86	0.91	0.87	0.90
benzofluorenes	0.77	0.74	0.77	0.73	0.80	0.76	0.79	0.75	0.80	0.76	0.80	0.77	0.79	0.76	0.76	0.73
benzoquinones	0.04	0.04	-0.0	-0.3	0.03	0.03	-0.0	-0.0	0.00	-0.0	0.08	0.08	0.09	0.08	0.06	0.02
carotenoids	0.79	0.76	0.77	0.74	0.79	0.76	0.79	0.75	0.79	0.75	0.76	0.72	0.76	0.69	0.73	0.68
chalcones	0.98	0.97	0.94	0.92	0.97	0.93	0.98	0.95	0.95	0.94	0.98	0.96	0.95	0.92	0.96	0.94
flavonoids	0.78	0.86	0.94	0.94	0.90	0.92	0.89	0.92	0.91	0.93	0.84	0.90	0.90	0.94	0.93	0.95
glutamine heterocycles	0.83	0.83	0.83	0.80	0.84	0.83	0.84	0.83	0.83	0.82	0.85	0.84	0.85	0.84	0.85	0.83
indole heterocycles	0.60	0.65	0.70	0.73	0.64	0.67	0.63	0.67	0.66	0.69	0.61	0.66	0.63	0.68	0.63	0.66
naphthoquinones	0.52	0.47	0.80	0.80	0.73	0.69	0.73	0.69	0.79	0.76	0.72	0.67	0.81	0.83	0.81	0.84
other heterocycles	0.66	0.80	0.69	0.81	0.65	0.80	0.66	0.80	0.64	0.78	0.62	0.76	0.62	0.75	0.63	0.75
other polyketides	0.89	0.72	0.87	0.88	0.77	0.80	0.76	0.79	0.80	0.83	0.77	0.81	0.86	0.89	0.88	0.92
perylenequinones	0.93	0.92	0.92	0.93	0.93	0.93	0.93	0.93	0.93	0.93	0.94	0.94	0.94	0.94	0.93	0.94
phenalenones	0.63	0.68	0.72	0.73	0.68	0.71	0.67	0.70	0.72	0.74	0.70	0.74	0.76	0.76	0.77	0.77
phenazines	0.76	0.82	0.78	0.85	0.78	0.84	0.78	0.84	0.79	0.85	0.78	0.85	0.71	0.86	0.79	0.86
phenoxazinones	0.45	0.35	0.44	0.14	0.47	0.28	0.48	0.27	0.46	0.23	0.46	0.29	0.49	0.20	0.50	0.17
polyenes	0.66	0.67	0.60	0.60	0.66	0.69	0.65	0.67	0.66	0.62	0.27	0.64	0.18	0.56	0.13	0.50
pterins / flavins	0.75	0.93	0.84	0.87	0.83	0.91	0.79	0.91	0.83	0.88	0.75	0.96	0.90	0.94	0.90	0.93
pulvinones	0.84	0.90	0.87	0.91	0.88	0.92	0.87	0.91	0.88	0.91	0.85	0.91	0.88	0.89	0.86	0.89
pyranones	0.39	0.40	0.40	0.39	0.40	0.39	0.40	0.39	0.42	0.39	0.41	0.41	0.41	0.40	0.41	0.40
pyridine heterocycles	0.88	0.93	0.97	0.96	0.96	0.95	0.96	0.96	0.96	0.95	0.98	0.96	0.98	0.97	0.98	0.97

pyrrole heterocycles	0.89	0.91	0.94	0.95	0.92	0.93	0.92	0.93	0.93	0.94	0.91	0.93	0.93	0.95	0.94	0.95
quinoline heterocycles	0.26	0.13	0.34	0.26	0.34	0.21	0.31	0.19	0.37	0.24	0.42	0.26	0.48	0.38	0.51	0.42
terphenyls	0.70	0.80	0.73	0.72	0.80	0.78	0.76	0.76	0.73	0.77	0.75	0.78	0.85	0.83	0.77	0.80
tyrosine heterocycles	0.60	0.87	0.69	0.81	0.69	0.87	0.65	0.88	0.70	0.86	0.56	0.85	0.59	0.78	0.60	0.77
xanthones	0.65	0.64	0.77	0.76	0.73	0.70	0.74	0.75	0.72	0.72	0.71	0.72	0.79	0.80	0.83	0.82

Table S3. In-class mean absolute errors (MAE) between experimental energies of the most intensive light absorption and vertical transition energies obtained by different methods for the natural colourants database. MAE is calculated after removal of systematic error for the whole database by application of linear regression. This table contains classes of colourants with more than 5 members.

Class	PBE0	PBE0 solv	ωB97XD4	ωB97XD4 solv	BMK	BMK solv	CAMB3LYP	CAMB3LYP solv	M06-2X	M06-2X solv	B2PLYP	B2PLYP solv	PBEQIHD	PBEQIHD solv	ωPBEPP86	ωPBEPP86 solv
angucyclinones	0.26	0.23	0.23	0.21	0.24	0.22	0.25	0.22	0.23	0.21	0.24	0.2	0.21	0.18	0.2	0.17
anthocyanins	0.18	0.29	0.13	0.21	0.15	0.26	0.16	0.26	0.13	0.23	0.12	0.21	0.06	0.11	0.06	0.11
anthraquinones	0.15	0.16	0.12	0.14	0.15	0.16	0.14	0.15	0.15	0.16	0.13	0.13	0.11	0.11	0.1	0.1
aurones	0.21	0.19	0.21	0.19	0.2	0.19	0.2	0.18	0.2	0.19	0.21	0.18	0.22	0.18	0.22	0.18
azaphilones	0.19	0.23	0.16	0.18	0.18	0.2	0.18	0.2	0.17	0.18	0.19	0.19	0.18	0.15	0.17	0.15
benzofluorenes	0.13	0.14	0.14	0.15	0.13	0.14	0.12	0.14	0.13	0.15	0.13	0.14	0.15	0.14	0.15	0.15
benzoquinones	0.3	0.32	0.36	0.37	0.3	0.3	0.3	0.32	0.3	0.29	0.3	0.31	0.31	0.27	0.36	0.28
carotenoids	0.14	0.18	0.16	0.18	0.2	0.23	0.16	0.19	0.18	0.21	0.05	0.09	0.06	0.06	0.08	0.07
chalcones	0.09	0.12	0.08	0.11	0.08	0.12	0.08	0.11	0.08	0.11	0.08	0.12	0.1	0.11	0.12	0.12
flavonoids	0.2	0.18	0.1	0.09	0.14	0.13	0.14	0.13	0.12	0.11	0.14	0.11	0.11	0.07	0.11	0.07
glutamine heterocycles	0.33	0.26	0.21	0.18	0.27	0.22	0.26	0.21	0.23	0.19	0.3	0.21	0.27	0.17	0.26	0.17
indole heterocycles	0.3	0.28	0.26	0.23	0.29	0.27	0.29	0.27	0.27	0.25	0.3	0.27	0.29	0.25	0.29	0.26
naphthoquinones	0.3	0.31	0.23	0.23	0.25	0.26	0.25	0.27	0.23	0.25	0.25	0.25	0.22	0.2	0.21	0.2
other heterocycles	0.34	0.33	0.36	0.3	0.34	0.31	0.34	0.3	0.35	0.3	0.35	0.29	0.38	0.31	0.4	0.32
other polyketides	0.2	0.24	0.19	0.19	0.22	0.22	0.22	0.21	0.2	0.2	0.21	0.2	0.2	0.16	0.21	0.17
perylenequinones	0.28	0.28	0.23	0.23	0.24	0.24	0.24	0.24	0.23	0.23	0.24	0.22	0.22	0.2	0.21	0.18
phenalenones	0.2	0.17	0.17	0.16	0.18	0.16	0.19	0.17	0.17	0.16	0.19	0.16	0.17	0.16	0.18	0.17

phenazines	0.33	0.32	0.31	0.27	0.32	0.29	0.31	0.29	0.31	0.27	0.31	0.27	0.32	0.24	0.3	0.24
phenoxazinones	0.14	0.15	0.17	0.19	0.14	0.16	0.15	0.17	0.15	0.17	0.14	0.15	0.13	0.16	0.13	0.17
polyenes	0.24	0.31	0.27	0.32	0.3	0.36	0.25	0.32	0.28	0.35	0.18	0.22	0.13	0.16	0.11	0.13
pterins / flavins	0.23	0.21	0.16	0.15	0.18	0.16	0.19	0.16	0.16	0.14	0.21	0.14	0.15	0.12	0.15	0.14
pulvinones	0.19	0.15	0.16	0.14	0.15	0.12	0.15	0.13	0.15	0.13	0.17	0.13	0.15	0.15	0.17	0.16
pyranones	0.2	0.19	0.18	0.19	0.19	0.18	0.18	0.18	0.18	0.18	0.19	0.18	0.2	0.18	0.21	0.18
pyridine heterocycles	0.14	0.22	0.13	0.15	0.15	0.18	0.14	0.18	0.15	0.17	0.14	0.16	0.1	0.11	0.08	0.09
pyrrole heterocycles	0.32	0.3	0.17	0.15	0.23	0.21	0.26	0.23	0.21	0.19	0.32	0.29	0.26	0.22	0.24	0.21
quinoline heterocycles	0.26	0.29	0.25	0.27	0.24	0.27	0.26	0.28	0.24	0.26	0.22	0.25	0.2	0.22	0.19	0.21
terphenyls	0.35	0.35	0.26	0.28	0.29	0.33	0.3	0.36	0.29	0.31	0.33	0.34	0.23	0.25	0.26	0.24
tyrosine heterocycles	0.36	0.35	0.3	0.27	0.32	0.31	0.33	0.32	0.3	0.27	0.43	0.33	0.39	0.3	0.38	0.3
xanthones	0.24	0.21	0.12	0.11	0.15	0.14	0.16	0.13	0.14	0.12	0.25	0.19	0.24	0.17	0.23	0.16

Table S4. In-class Pearson correlation coefficients for experimental extinction coefficients and oscillator strengths calculated through transition electric dipole moments. Green colour marks significant correlation (P-value < 0.05).

class	PBE0	PBE0 solv	ωB97XD4	ωB97XD4 solv	BMK	BMK solv	CAMB3LYP	CAMB3LYP solv	M06-2X	M06-2X solv	B2PLYP	B2PLYP solv	PBEQIHD	PBEQIHD solv	ωPBEPP86	ωPBEPP86 solv
angucyclinones	0.4	0.43	0.45	0.46	0.43	0.45	0.43	0.45	0.43	0.45	0.44	0.46	0.45	0.47	0.45	0.47
anthocyanins	-0.13	0.38	0.68	0.75	0.16	0.62	0.21	0.64	0.34	0.69	0.27	0.67	0.54	0.72	0.64	0.74
anthraquinones	0.15	-0.03	0.29	0.03	0.14	-0.2	0.15	-0.20	-0.10	0.04	-0.14	-0.20	-0.06	-0.04	0.05	-0.03
aurones	0.6	0.46	0.6	0.44	0.52	0.42	0.13	0.42	0.51	0.43	0.62	0.40	0.58	0.41	0.27	0.42
azaphilones	-0.44	-0.41	-0.45	-0.44	-0.47	-0.45	-0.46	-0.45	-0.46	-0.44	-0.47	-0.46	-0.37	-0.47	-0.37	-0.48
benzoquinones	0.52	0.49	0.58	0.55	0.51	0.51	0.53	0.52	0.51	0.52	0.5	0.5	0.51	0.52	0.56	0.54
carotenoids	-0.34	-0.37	-0.36	-0.38	-0.35	-0.37	-0.35	-0.37	-0.35	-0.37	-0.36	-0.38	-0.36	-0.38	-0.36	-0.38
glutamine heterocycles	-0.13	-0.83	-0.81	-0.83	-0.81	-0.84	-0.81	-0.83	-0.81	-0.83	-0.84	-0.86	-0.84	-0.87	-0.85	-0.88
indole heterocycles	0.45	0.45	0.47	0.51	0.45	0.48	0.46	0.49	0.45	0.51	0.45	0.50	0.46	0.50	0.44	0.49
naphthoquinones	0.35	0.37	0.31	0.36	0.29	0.35	0.32	0.34	0.35	0.31	0.34	0.30	0.37	0.37	0.38	0.38
other heterocycles	0.44	0.62	0.48	0.53	0.46	0.71	0.46	0.63	0.62	0.54	0.45	0.64	0.46	0.5	0.68	0.49
other polyketides	0.17	0.08	0.02	0.08	0.07	0.11	0.01	0.09	0.01	0.09	0.05	0.12	0.08	0.14	0.09	0.15
perylenequinones	0.72	0.73	0.44	0.41	0.59	0.57	0.6	0.6	0.54	0.58	0.53	0.52	0.41	0.4	0.35	0.34
phenalenones	0.52	0.53	0.5	0.44	0.55	0.53	0.54	0.52	0.5	0.47	0.56	0.54	0.55	0.52	0.54	0.49
phenazines	0.27	0.35	0.32	0.28	0.34	0.33	0.36	0.29	0.37	0.33	0.34	0.33	0.21	0.31	0.35	0.29
phenoxazinones	0.25	0.28	0.04	0.52	0.1	0.49	0.39	0.26	-0.23	0.46	-0.04	0.31	0.12	0.6	0.45	0.61
pulvinones	0.39	0.48	-0.08	0.66	0.53	0.69	0.54	0.7	0.43	0.68	0.43	0.67	0.37	0.62	0.63	0.61
pyranones	0.35	0.34	0.43	0.4	0.48	0.37	0.58	0.38	0.63	0.38	0.40	0.39	0.41	0.40	0.41	0.41
pyridine heterocycles	-0.06	0.53	0.53	0.53	0.53	0.7	0.48	0.71	0.53	0.53	0.52	0.52	0.52	0.54	0.52	0.55
pyrrole heterocycles	0.37	0.49	0.44	0.5	0.45	0.52	0.44	0.52	0.45	0.52	0.46	0.51	0.44	0.49	0.44	0.48
quinoline heterocycles	-0.28	-0.25	-0.26	-0.19	-0.02	-0.15	-0.33	-0.21	-0.09	-0.20	-0.05	-0.33	-0.20	-0.30	-0.20	-0.29
terphenyls	0.8	0.51	0.03	0.33	0.45	0.26	0.46	0.28	-0.12	0.3	-0.03	0.33	0.04	0.45	-0.04	0.45

xanthones	-0.18	-0.08	-0.07	-0.04	-0.18	-0.08	-0.18	-0.09	-0.15	-0.09	-0.10	-0.03	-0.03	0.02	-0.02	0.03

Table S5. Pearson correlation coefficients (r) of quantum chemical descriptors with experimental the lowest and the most intensive energy of light absorption (when expressed in wavelengths is called λ_{max}). Green colour marks significant correlation (P-value < 0.05).

Descriptor	Full database (gas)	Full database (solvent)	Indole heterocycles (gas)	Indole heterocycles (solvent)	AQs (gas)	AQs (solvent)
HOMO	-0.13	-0.31	-0.33	-0.42	-0.66	-0.74
LUMO	0.41	0.51	0.24	0.42	0.48	0.48
Dipole moment (μ)	-0.04	-0.04	-0.02	-0.02	-0.07	-0.07
Electronegativity (χ)	-0.15	-0.11	0.041	-0.03	0.30	0.50
Hardness (η)	0.71	0.71	0.69	0.71	0.88	0.86
Electrophilicity (ω)	-0.30	-0.38	-0.06	-0.27	-0.22	-0.11
Mean polarisability (α)	-0.27	-0.31	-0.35	-0.39	-0.21	-0.23
Anisotropic polarisability ($\Delta\alpha$)	-0.24	-0.29	-0.42	-0.50	-0.41	-0.59

Table S6 Covariation matrix of computed quantum chemical descriptors based on the whole dataset of natural colourants. Green colour marks pairs of descriptors with significant correlation (P-value < 0.05).

	HOMO	LUMO	μ	χ	η	ω	α	$\Delta\alpha$
HOMO	1.0	0.70	0.07	-0.91	-0.29	-0.73	0.41	0.39
LUMO	0.70	1.0	0.03	-0.92	0.48	-0.92	-0.06	-0.09
μ	0.07	0.03	1.0	-0.05	-0.05	0.01	0.01	-0.07

χ	-0.91	-0.92	-0.05	1.0	-0.11	0.91	-0.17	-0.14
η	-0.29	0.48	-0.05	-0.11	1.0	-0.32	-0.60	-0.60
ω	-0.73	-0.92	0.01	0.91	-0.32	1.0	-0.04	-0.01
α	0.41	-0.06	0.01	-0.17	-0.60	-0.04	1.0	0.88
$\Delta\alpha$	0.39	-0.09	-0.07	-0.14	-0.60	-0.01	0.88	1.0

Table S7. Regression equations built for different subsets of molecules using "elastic net" linear regression where α and γ are coefficients at L_1 and L_2 penalties in the loss function which shows the lowest error on the cross-validation set.

Set	Equation (λ_{max} in eV)	α	γ
Full (gas)	$\lambda_{max} = -1.35 + 5.90 * 10^{-3} * LUMO + 5.53 * 10^{-1} * \eta + \ldots$ $\ldots + 4.21 * 10^{-3} * \mu - 2.52 * 10^{-5} * \alpha + 4.68 * 10^{-4} * \Delta\alpha$	4.1e-3	1.0
Full (solvent)	$\lambda_{max} = -1.62 - 6.95 * 10^{-2} * HOMO + 0.53 * \eta - 1.32 * 10^{-3} * \mu - \ldots$ $\ldots -2.31 * 10^{-4} * \alpha + 3.13 * 10^{-4} * \Delta\alpha$	4.2e-4	0.0
Indole heterocycles (gas)	$\lambda_{max} = -0.87 - 2.76 * 10^{-2} * HOMO + 0.49 * \eta + 2.33 * 10^{-3} * \mu - \ldots$ $\ldots -1.42 * 10^{-4} * \alpha - 8.94 * 10^{-5} * \Delta\alpha$	1.5e-2	0.4
Indole heterocycles (solvent)	$\lambda_{max} = -1.59 + 0.60 * \eta + 0.02 * \mu + 9.01 * 10^{-4} * \alpha + 2.11 * 10^{-3} * \Delta\alpha$	1.3e-2	1.0
AQ (gas)	$\lambda_{max} = -3.54 + 1.29 * HOMO + 15.33 * LUMO - 2.10 * \chi \ldots$ $\ldots +2.66 * 10^{-3} * \eta + 28.17 * \omega - 2.27 * 10^{-2} * \mu + \ldots$ $\ldots +6.90 * 10^{-4} * \alpha + 1.41 * 10^{-3} * \Delta\alpha$	9.3e-3	1.0
AQ (solvent)	$\lambda_{max} = -1.33 - 0.25 * HOMO + 7.49 * 10^{-2} * LUMO + \ldots$	1e-5	0.96

	$\ldots +8.87 * 10^{-2} * \chi + 0.33 * \eta - 0.53 * \omega + \ldots$ $\ldots -3.00 * 10^{-3} * \mu + 4.20 * 10^{-5} * \alpha + 3.45 * 10^{-4} * \Delta\alpha$		

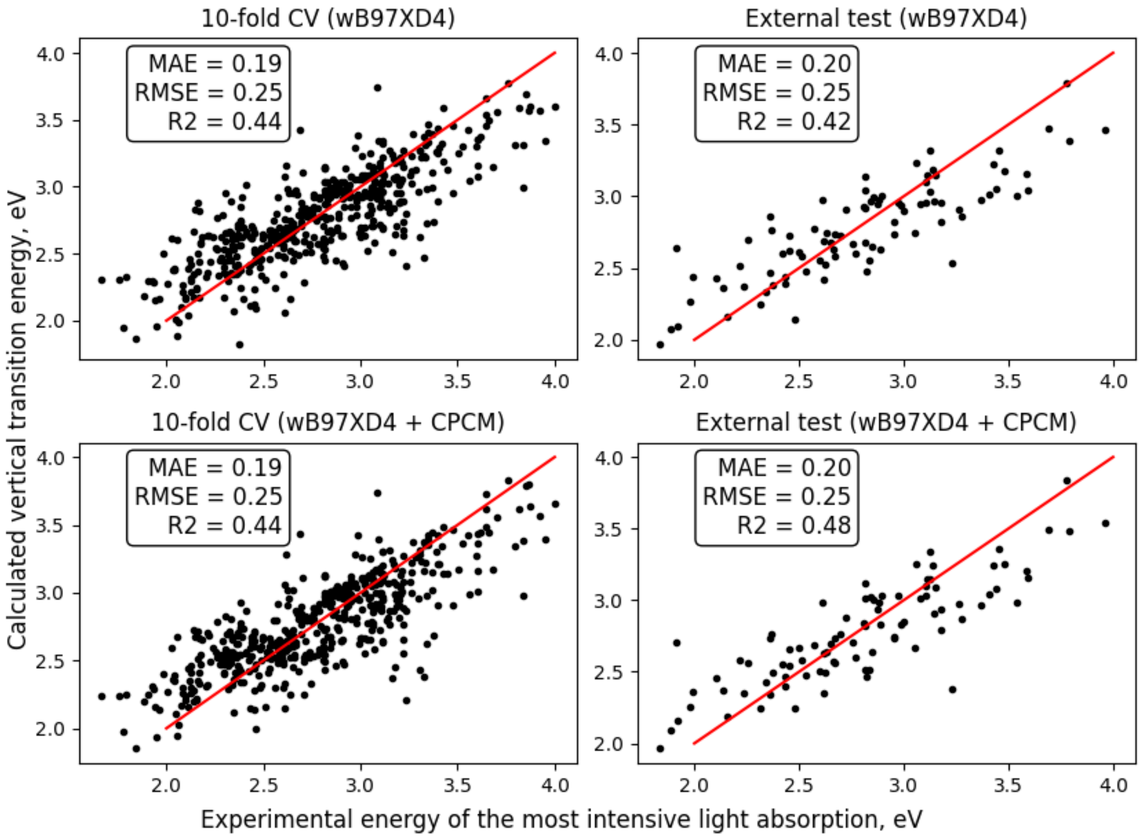

Figure S3. Performance of a hybrid approach combining linear regression with descriptors calculated using quantum mechanical descriptors (hardness, electrophilicity, dipole moment, polarizability, etc.). (Left) 10-fold cross-validation (applied for the training set after random train/test split) results for the whole database using descriptors calculated in vacuum (top) and using CPCM solvation model (bottom). (Right) test set performance for the parameter set chosen using cross-validation.

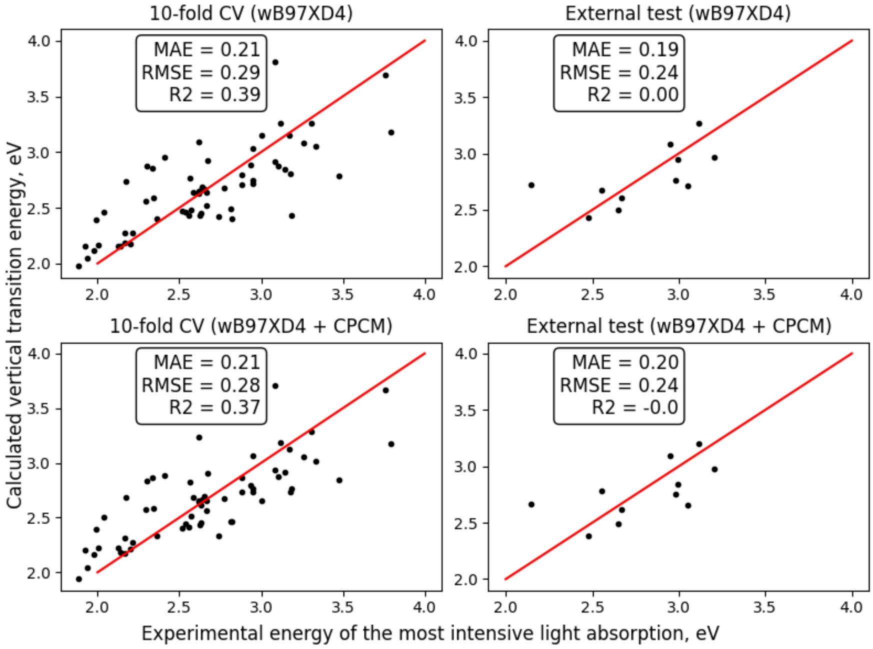

Figure S4. Performance of a hybrid approach combining linear regression with descriptors calculated using quantum mechanical descriptors (hardness, electrophilicity, dipole moment, polarizability, etc.). (Left) 10-fold cross-validation (applied for the training set after random train/test split) results for the for a subset of the original database composed of indole-based heterocycles using descriptors calculated in vacuum (top) and using CPCM solvation model (bottom). (Right) test set performance for the parameter set chosen using cross-validation.

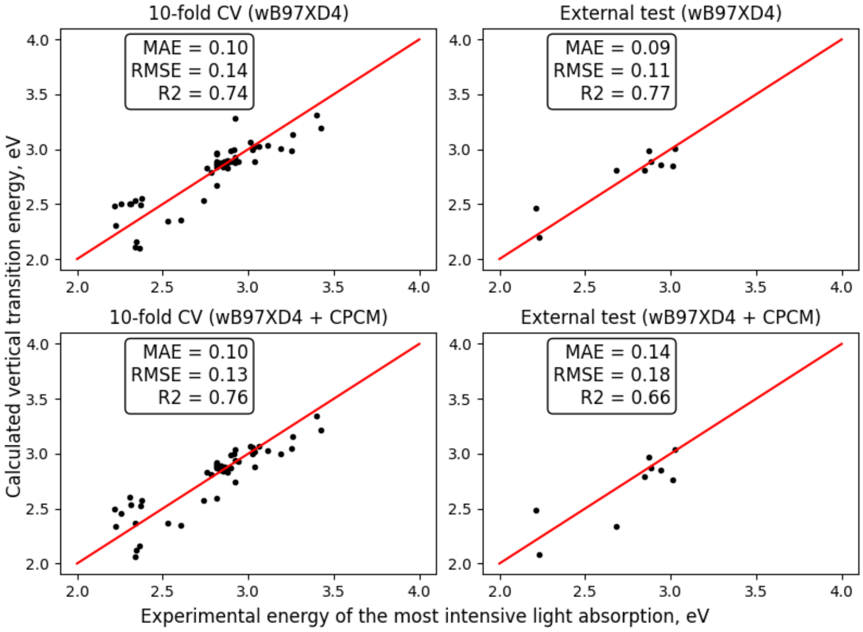

Figure S5. Performance of a hybrid approach combining linear regression with descriptors calculated using quantum mechanical descriptors (hardness, electrophilicity, dipole moment, polarizability, etc.). (Left) 10-fold cross-validation (applied for the training set after random train/test split) results for the for a subset of the original database composed of anthraquinone derivatives using descriptors calculated in vacuum (top) and using CPCM solvation model (bottom). (Right) test set performance for the parameter set chosen using cross-validation.

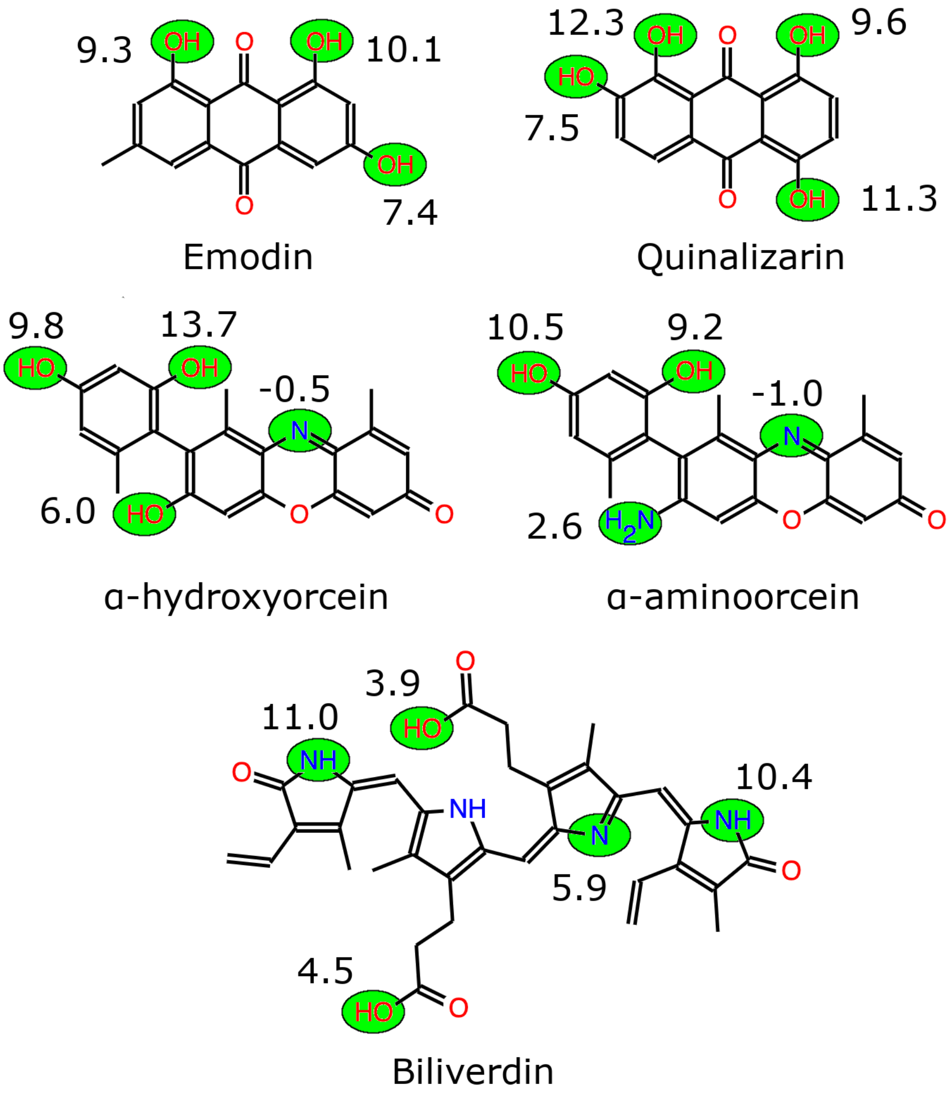

Figure S6. pKa values predicted using ChemAxon for colourants tested experimentally in this work.

Table S8. Calculated transition energies (before and after systematic error removal) and oscillator strengths for emodin, quinalizarin, α-hydroxyorcein, and α-aminoorcein investigated in this work at ωB97X-D4/def2-TZVP/CPCM(water), ma-def2-TZVP basis set was used for anions. Biliverdin was calculated at ωB97X-D4/def2-SVP/CPCM(water), ma-def2-SVP due to long SCF convergence times. Microspecies labels are assigned to structures in Schemes 4-8. All transitions close to visible spectral range and having oscillator strength more than 0.01 are reported.

colourant	Microspecies	Calculated transition energies, eV	Calculated transition energies (after systematic error correction), eV	Calculated oscillator strength
Emodin	S000	2.62; 3.30; 3.37; 3.62	2.13; 2.81; 2.86; 3.04	0.26; 0.03, 0.02; 0.10
	S100	2.76; 3.35	2.41; 2.85	0.28; 0.04
	S010	2.65; 3.42; 3.47	2.34; 2.90; 2.93	0.26; 0.10; 0.03

	S110	2.92; 3.98	2.53; 2.85	0.28; 0.12
	S111	3.44	2.91	0.35
Quinalizarin	S0000	2.42; 3.22	2.17; 2.75	0.62; 0.07
	S0100	2.53; 3.56	2.25; 3.00	0.51, 0.10
	S0001	2.38	2.14	0.69
	S0101	2.64; 3.20	2.33; 2.73	0.61; 0.10
	S0110	2.62; 3.33	2.31; 2.83	0.41; 0.05
	S0111	2.60; 3.43	2.30; 2.90	0.44; 0.12
	S1111	3.12; 3.72	2.70; 3.12	0.42; 0.06
α-hydroxyorcein	S0000	2.46; 2.80	2.20; 2.44	0.86; 0.31
	S0100	2.53	2.25	1.19
	S1100	2.62	2.31	1.15
	S1101	3.11	2.68	0.85
	S1111	2.61; 3.28	2.30; 2.79	0.59; 0.28
α-aminoorcein	S0000	2.72; 3.00	2.38; 2.59	1.05; 0.03
	S1000	2.77	2.44	1.11
	S0100	2.73	2.39	1.09
	S1100	2.87	2.49	1.07
	S1101	3.28	2.80	0.52
	S1111	2.75; 3.09	2.41; 3.03	0.24; 0.56
Biliverdin	S00000	2.266; 3.215; 3.598	2.053; 2.747; 3.027	1.37; 0.51; 0.14
	S10000	2.297; 3.332; 3.635	2.076; 2.832; 3.054	0.58; 0.78; 0.59
	S00001	2.159; 3.318; 3.783; 3.886	1.975; 2.822; 3.162; 3.238	1.98; 0.04; 0.24; 0.15
	S10001	2.570; 3.697; 3.809	2.276; 3.100; 3.181	1.89; 0.22; 0.29
	S10011	2.263; 3.681; 4.034	2.051; 3.088; 3.346	1.69; 0.04; 0.27
	S10111	2.188; 3.717; 3.942	1.996; 3.114; 3.279	1.26; 1.20; 0.17

	S11011	2.087; 3.593; 3.974	1.922; 3.023; 3.302	1.74; 0.05; 0.10
	S11111	2.234; 3.787	2.030; 3.165	0.96; 1.67

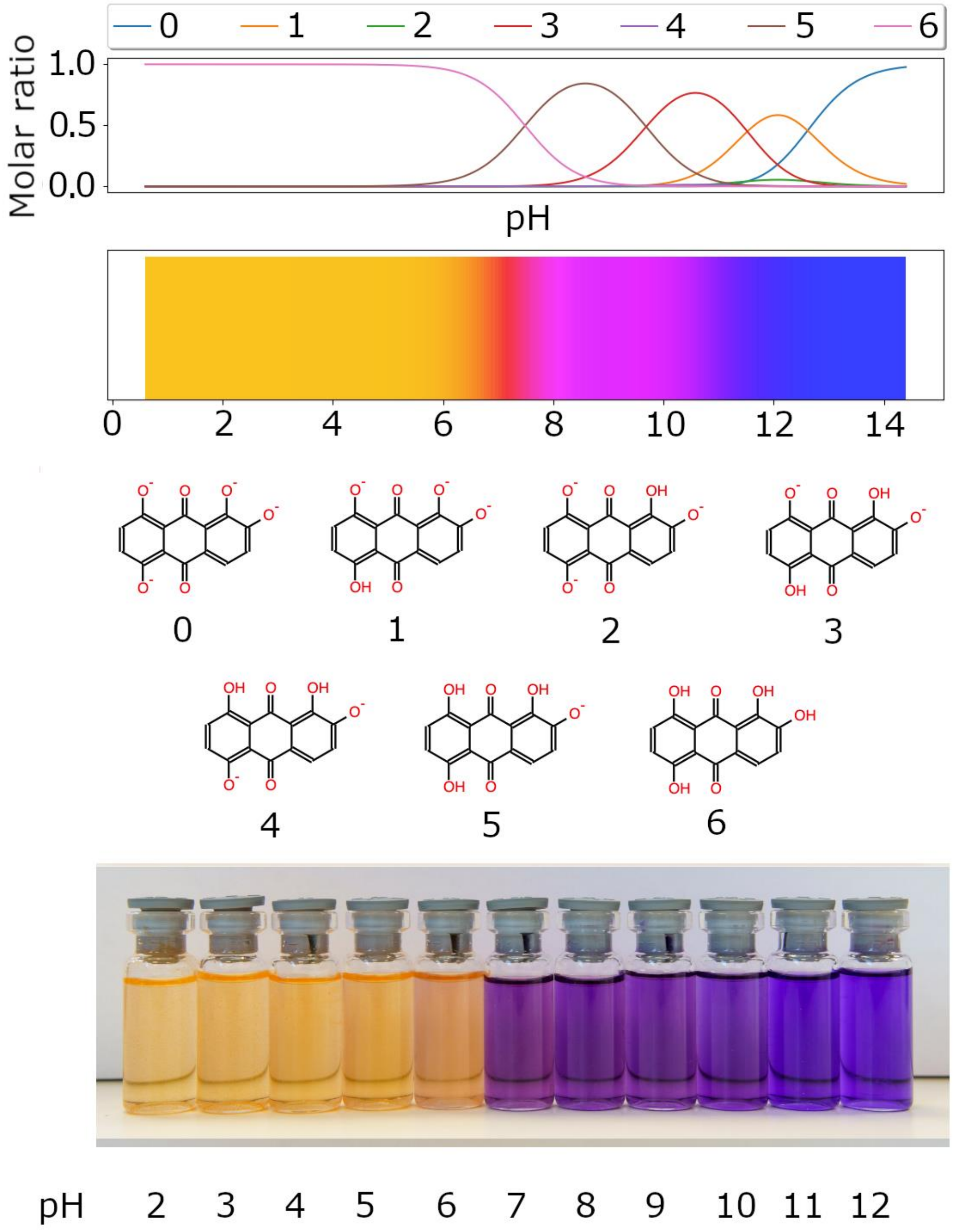

Figure S7. Predicted (top) vs experimental colour (bottom) for quinalizarin solution water-methanol mixture at different pH levels (2-12). Predicted colour is calculated based on molar ratios (top graph) of coloured protonated/deprotonated species (numbers in the legend are related to structure numbers).

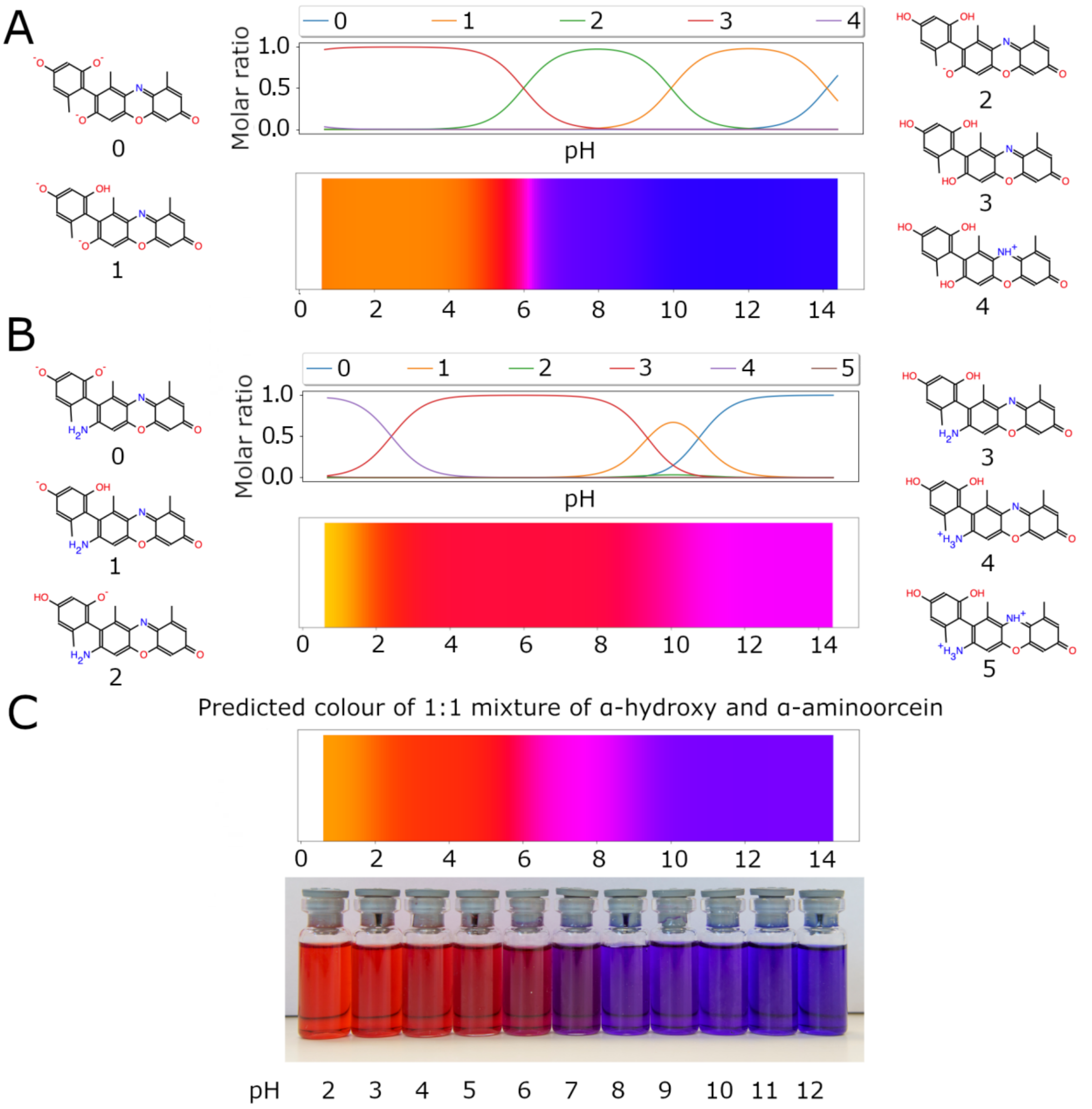

Figure S8. Predicted (top) vs experimental colour (bottom) for orcein solution water-methanol mixture at different pH levels (2-12): (A) α-hydroxy-orcein; (B) α-amino-orcein. Predicted colour is calculated based on molar ratios (top graph) of coloured protonated/deprotonated species (numbers in the legend are related to structure numbers). (C) Prediction of colour with an approximation that orcein is 1:1 mixture of α-amino- and α-hydroxy-orcein.

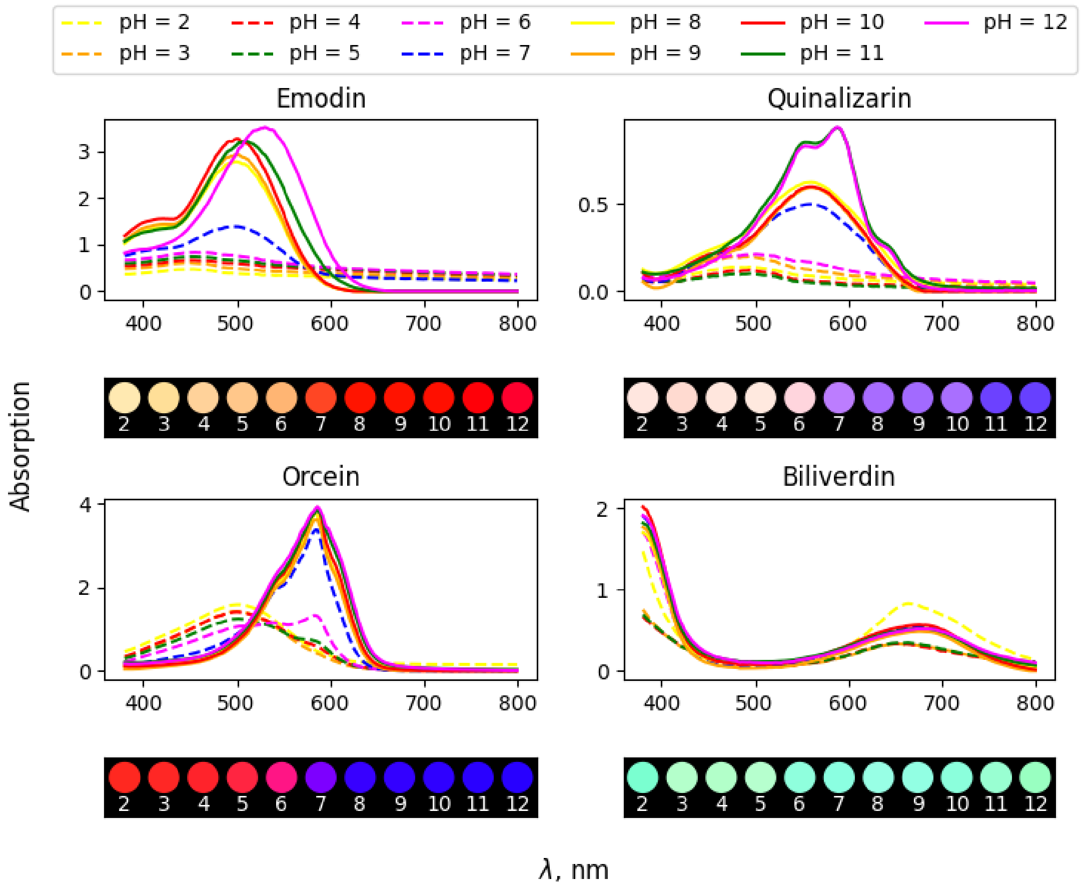

Figure S9. Experimental absorption spectra (wavelength vs optical density) with corresponding derived colours obtained for emodin, quinalizarin, orcein, and biliverdin at pH range from 2 to 12. Emodin and quinalizarin demonstrate relatively high background signals at acidic pH due to scattering of colloidal dye particles.

Table S9. Experimental colours obtained from absorption spectra of model colourants and predicted colour based on simulated absorption spectra using gaussian approximation in Lab colour space (Gimp 2.10 - Lab values were obtained from a colour picker tool). Euclidean distances are calculated to mark differences.

colourant	pH	Experimental colour			Predicted colour			Euclidean distance
		L	*a	*b	L	*a	*b	
Emodin	2	92.9	-0.6	30.0	96.5	-24.5	80.9	56.3
	3	90.0	1.6	38.9	96.5	-24.5	80.9	49.9
	4	86.8	8.5	33.7	96.5	-24.5	80.9	58.4
	5	83.9	12.6	38.1	96.6	-23.7	81.1	57.7
	6	79.4	19.9	44.1	93.5	-14.4	80.3	51.8
	7	57.8	67.9	59.0	65.8	39.5	68.9	31.1
	8	53.8	78.5	67.4	59.8	57.6	36.7	37.6
	9	53.7	78.6	67.4	56.2	77.4	-9.1	76.6
	10	53.6	79.0	67.3	59.5	86.9	-48.9	116.6
	11	53.3	80.1	65.2	60.2	86.3	-49.4	115.0
	12	53.5	80.7	50.5	60.3	86.0	-49.3	100.2
Quinalizarin	2	93.1	7.4	6.5	81.8	6.0	79.4	73.8
	3	89.9	11.3	9.7	81.8	6.0	79.4	70.4
	4	93.1	7.1	7.5	81.4	6.1	79.1	72.6
	5	93.8	5.9	7.6	81.2	6.6	79.1	72.6
	6	89.0	15.9	1.6	77.0	15.1	75.1	74.5
	7	63.8	48.9	-55.8	56.9	63.2	48.1	105.1
	8	58.3	53.1	-64.7	60.8	88.1	-55.1	36.4
	9	57.3	52.8	-66.4	57.3	88.7	-66.0	35.9
	10	59.0	51.7	-63.6	55.8	88.8	-68.4	37.5
	11	44.5	64.3	-87.4	44.2	78.8	-87.8	14.5
	12	43.9	64.2	-88.4	39.3	68.3	-96.1	9.9
Orcein	2	55.0	75.8	58.4	63.0	57.5	72.6	24.50
	3	54.9	76.1	56.1	57.0	73.4	69.1	13.4
	4	54.7	76.9	53.1	56.2	75.5	68.0	15.0
	5	55.0	77.2	41.7	55.1	78.8	63.7	22.0
	6	55.6	83.3	2.1	55.4	83.2	13.3	11.2
	7	40.5	83.0	-93.9	59.1	91.3	-49.4	48.9
	8	34.1	79.8	-104.9	58.2	91.6	-63.8	49.0
	9	33.8	79.7	-105.3	47.4	81.4	-81.8	27.2
	10	33.6	79.6	-105.6	39.8	75.0	-94.7	13.3
	11	33.4	79.5	-106.0	38.4	74.0	-97.0	11.7
	12	33.3	79.5	-106.1	38.3	73.9	-97.3	11.6
Biliverdin (single conformation)	2	91.8	-47.5	11.4	90.2	-57.0	18.3	11.8
	3	94.1	-33.3	17.6	91.2	-48.1	-4.8	27.06
	4	94.0	-34.3	18.4	30.5	67.5	-110.5	176.1
	5	94.3	-32.3	16.8	30.2	68.6	-111.0	175.0
	6	92.8	-39.9	6.3	30.2	68.6	-111.0	171.6
	7	92.8	-39.9	4.1	31.8	63.8	-108.2	164.6
	8	93.4	-35.9	3.0	32.6	61.6	-106.9	159.0
	9	93.1	-37.8	3.6	33.6	59.1	-105.3	157.4
	10	92.7	-41.0	6.5	40.6	40.9	-93.7	139.5

	11	93.0	-39.6	12.2	57.4	5.5	-66.3	97.3
	12	92.6	-42.9	20.5	57.9	4.4	-65.4	104.0
Biliverdin (conformational ensemble)	2	91.8	-47.5	11.4	88.0	-77.3	68.7	64.7
	3	94.1	-33.3	17.6	88.2	-75.4	58.7	59.1
	4	94.0	-34.3	18.4	88.9	-67.7	28.9	35.4
	5	94.3	-32.3	16.8	89.5	-61.9	12.1	30.4
	6	92.8	-39.9	6.3	89.5	-61.8	11.6	22.7
	7	92.8	-39.9	4.1	89.4	-62.9	14.5	25.5
	8	93.4	-35.9	3.0	89.4	-62.9	14.5	29.6
	9	93.1	-37.8	3.6	90.0	-57.1	1.0	19.7
	10	92.7	-41.0	6.5	64.6	1.8	-54.7	79.8
	11	93.0	-39.6	12.2	49.8	71.6	-77.9	149.5
	12	92.6	-42.9	20.5	48.2	72.6	-80.6	159.8
Average Euclidean distance (excluding Biliverdin (single conformation)):								**50.9**

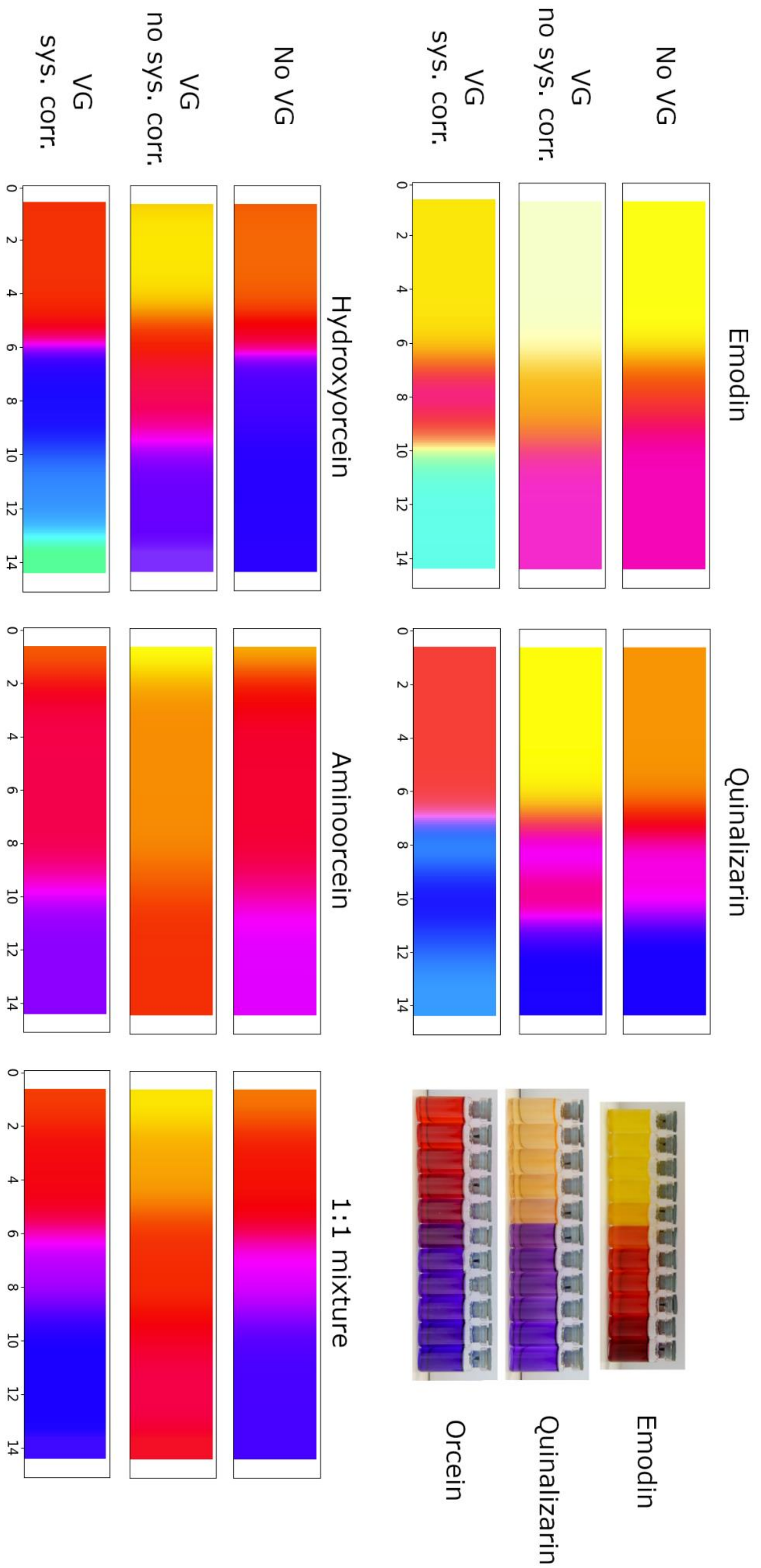

Figure S10. Comparison of line shape approximation techniques and their influence on colour prediction. “No VG“ - Gaussian lineshape function centred on the calculated vertical transition (systematic error corrected) without consideration of vibronic transitions. “VG no sys. Corr.” - spectra calculated by ORCA are convoluted with a gaussian function (with σ = 0.08 eV) for additional spectral broadening the converted to colour. “VG sys. corr.” - the same approach

as before but all absorption spectra are shifted using the previously defined systematic error correction.

Table S10. Results of TD-DFT calculations (ωB97X-D4/def2-SVP(CPCM: MeOH)) after removal of the systematic error for the generated bikaverin derivatives. Only structures with absorption energies predicted by ML-model below 2.4 eV are shown. The colour is predicted for a 100 μM solution in MeOH. Bikaverin ethanolamine conjugates with predicted blue colour are characterized by ground state electronic energies with zero-point energy (ZPE) correction.

#	Structure	Predicted colour			Absorption energies, eV	Oscillator strengths
		L	*a	*b		
1	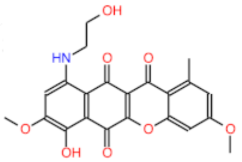<br>Relative energy: 2.5 kcal/mol	49.8	32.5	-78.9	2.13	0.29
2	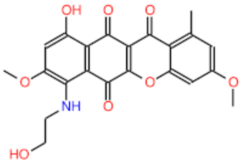<br>Relative energy: 1.7 kcal/mol	40.74	57.5	-93.8	2.17; 2.97	0.39; 0.01
3	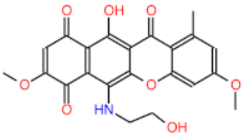<br>Relative energy: 0.0 kcal/mol	40.8	60.7	-93.7	2.18; 3.02	0.36; 0.03
4	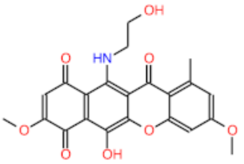<br>Relative energy: 9.7 kcal/mol	64.4	19.3	-55.1	2.18; 2.71; 2.82; 2.98; 3.09	0.25; 0.03; 0.03; 0.05; 0.02
5	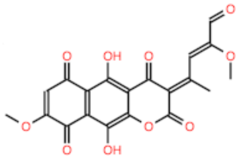	60.5	87.6	-40.9	2.38; 2.98	0.32; 0.02
6	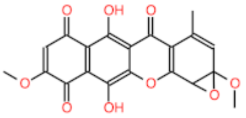	64.0	82.1	-44.5	2.28; 2.76; 3.10	0.24; 0.06; 0.10

7	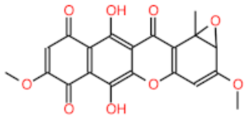	60.2	77.0	-13.4	2.41; 2.75; 3.03	0.30; 0.02; 0.06
8	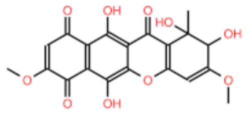	62.7	62.9	14.0	2.46; 2.54; 2.90; 3.05	0.29; 0.04; 0.02; 0.06
9	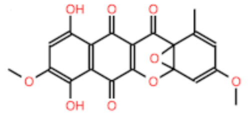	52.4	82.3	-74.0	2.30	0.24
10	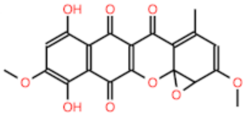	62.2	72.5	-13.4	2.29; 2.85; 2.90; 2.94; 3.03	0.25; 0.08; 0.07; 0.04; 0.10
11	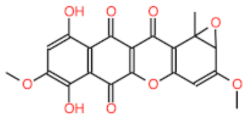	51.9	83.8	-74.9	2.30; 3.16	0.26; 0.05
12	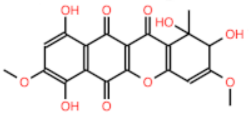	56.6	85.7	-67.2	2.30; 3.00; 3.02;	0.27; 0.04; 0.06
13	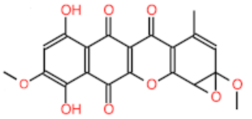	53.0	84.2	-73.1	2.30; 2.96	0.27; 0.02
14	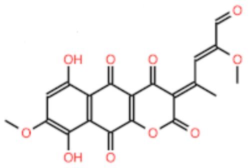	60.3	79.8	-21.9	2.36; 2.71; 3.05; 3.13	0.25; 0.03; 0.05; 0.10
15	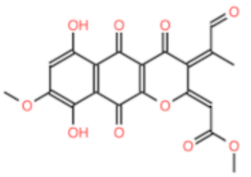	40.7	57.5	-93.8	2.26; 2.47; 2.65; 2.79; 3.08	0.24; 0.02; 0.01; 0.09; 0.19
16	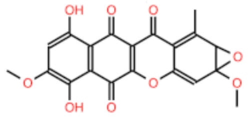	59.1	81.1	-18.0	2.35; 2.90; 3.04	0.30; 0.10; 0.12

Supplementary References

1. Sun, Q. *et al.* Recent developments in the PySCF program package. *J. Chem. Phys.* **153**, 024109 (2020).
2. Kazakova, O., Lipkovska, N. & Barvinchenko, V. Keto-enol tautomerism of curcumin in the preparation of nanobiocomposites with fumed silica. *Spectrochim. Acta. A. Mol. Biomol. Spectrosc.* **277**, 121287 (2022).
3. Boer, J. J. de, Bright, D., Dallinga, G. & G. Hewitt, T. Crystal and molecular structure of the chloroform solvate of bikaverin. *J. Chem. Soc. C Org.* **0**, 2788–2791 (1971).
4. International Commission On Illumination (Cie). CIE standard illuminant D65. https://cie.co.at/datatable/cie-standard-illuminant-d65.
5. RGB/XYZ Matrices. http://www.brucelindbloom.com/index.html?Eqn_RGB_XYZ_Matrix.html.
6. Turro, N., Ramamurthy, V. & Scaiano, J. *Modern Molecular Photochemistry of Organic Molecules*. (University Science Books, Sausalito).
7. P. Greenman, K., H. Green, W. & Gómez-Bombarelli, R. Multi-fidelity prediction of molecular optical peaks with deep learning. *Chem. Sci.* **13**, 1152–1162 (2022).
8. Joung, J. F. *et al.* Deep Learning Optical Spectroscopy Based on Experimental Database: Potential Applications to Molecular Design. *JACS Au* **1**, 427–438 (2021).
9. Bannwarth, C., Ehlert, S. & Grimme, S. GFN2-xTB—An Accurate and Broadly Parametrized Self-Consistent Tight-Binding Quantum Chemical Method with Multipole Electrostatics and Density-Dependent Dispersion Contributions. *J. Chem. Theory Comput.* **15**, 1652–1671 (2019).
