# Supplementary material for: DyeDactic workflow to predict halochromism of biosynthetic colourants
Source: Commun Chem. 2026 Jan 10;9:79. doi: 10.1038/s42004-025-01881-9 (PMC12894750; doi:10.1038/s42004-025-01881-9)
Supplement: Supplementary file 2 — Description of Additional Supplementary Files [file 42004_2025_1881_MOESM2_ESM.pdf]

Description of Additional Supplementary Files:

File name: Supplementary Data 1

Description: Source data underlying the graphs and charts presented in the main figures.
